# Population of Monocyte-Derived Macrophages

**DOI:** 10.1016/j.immuni.2015.01.018

**Published:** 2015-02-17

**Authors:** Naomi McGovern, Andreas Schlitzer, Merry Gunawan, Laura Jardine, Amanda Shin, Elizabeth Poyner, Kile Green, Rachel Dickinson, Xiao-nong Wang, Donovan Low, Katie Best, Samuel Covins, Paul Milne, Sarah Pagan, Khadija Aljefri, Martin Windebank, Diego Miranda-Saavedra, Anis Larbi, Pavandip Singh Wasan, Duan Kaibo, Michael Poidinger, Venetia Bigley, Florent Ginhoux, Matthew Collin, Muzlifah Haniffa

(Immunity *41*, 465–477, September 18, 2014)

## Main Text

In the original publication of this article, the name of an author was inadvertently misspelled. The corrected author name is Diego Miranda-Saavedra. The spelling is now correct in the online version of the study. The authors apologize for the inconvenience.

