# Supplementary material for: Human Dermal CD14+ Cells Are a Transient Population of Monocyte-Derived Macrophages
Source: Immunity. 2014 Sep 18;41(3):465–77. doi: 10.1016/j.immuni.2014.08.006 (PMC4175180; doi:10.1016/j.immuni.2014.08.006)
Supplement: Document S1. Figures S1–S2, Tables S1–S2, and Supplemental Experimental Procedures [file mmc1.pdf]

Immunity, Volume *41*

Supplemental Information

Human Dermal $CD14^+$ Cells Are a Transient Population of Monocyte-Derived Macrophages

Naomi McGovern, Andreas Schlitzer, Merry Gunawan, Laura Jardine, Amanda Shin, Elizabeth Poyner, Kile Green, Rachel Dickinson, Xiao-nong Wang, Donovan Low, Katie Best, Samuel Covins, Paul Milne, Sarah Pagan, Khadija Aljefri, Martin Windebank, Diego Miranda Saavedra, Pavandip Singh Wasan, Duan Kaibo, Michael Poidinger, Venetia Bigley, Florent Ginhoux, Matthew Collin, and Muzlifah Haniffa

Supplemental Experimental Procedures

Microarray analysis

Microarray data from purified dermal macrophages, epidermal LCs and $CD14^+$ cells hybridized on Illumina Human WG-6 BeadChip were integrated with GSE35457 Illumina HumanHT-12 V4.0 microarray data of human blood and skin monocyte and DC subsets. The integrated data were quantile normalized without background correction. Tissue specific probes were determined with samples from GSE35457 dataset using a t-test step similar to that previously described (Haniffa et al., 2012) but without using the Tissue-specific Gene Expression and Regulation (TiGER) database (Liu et al., 2008). Tissue specific probes were removed from the integrated data. Finally, ComBat (Johnson et al., 2007) was used to correct the batch effect observed in the integrated data. Signature genes for each cell subtype were identified by comparing one subtype versus other subtypes pooled using t-test and selected with Benjamini-Hochberg (BH) multiple testing (Benjamini and Hochberg, 1995) corrected p-value of < 0.05 and fold-change of > 1.5.

To identify the mouse homolog of dermal $CD14^+$ cells, human APC subsets were compared with mouse APC subsets in mouse microarray data GSE49358 (Affymetrix Mouse Gene 1.0 ST array). Mouse array data was processed using Bioconductor *oligo* pacakge (http://www.bioconductor.org) with quantile normalization and gene level expression value summarization using only the core probes. For genes with multiple transcript clusters mapping, the average expression values of the transcript clusters were used. For comparison of human APC subsets to mouse APC subsets, signatures of human APC subsets were first converted to mouse signatures by using HomoloGene table build 67 (http://www.ncbi.nlm.nih.gov/homologene/) for ortholog gene mapping. Enrichment scores were then computed on the mouse APC subsets with the converted mouse signatures using connectivity map (CMAP) analysis as previously described (Haniffa et al., 2012).

For comparative analysis of genes defining monocyte-macrophages in contrast to DCs between human and mouse, the up-regulated and down-regulated genes comparing monocyte-mocrophages to myeloid DC subsets were identified in human array data and mouse array data separately. For human array data, the monocyte, macrophage, and $CD14^+$ DC samples were pooled and compared to other DC subsets. For mouse data, monocyte, macrophage and DC subsets from the ImmGen data GSE15907 ($DC.8^+$ Sp, $DC4^+$ Sp, $DC.8^+$ SLN, $DC.4^+$ SLN,

DC.IIhilang^{+}103^{+}11blo.SLN, DC.IIhilang^{-}103^{-}11b^{+}.SLN, DC.103^{+}11b^{-}.Lu, DC.103^{-}11b^{+}24^{+}.Lu, Mo.6C^{+}II^{-}.Bl, Mo.6C^{+}II^{+}.Bl, Mo.6C^{-}II^{-}.Bl, Mo.6C^{-}II^{+}.Bl, Mo.6C^{-}IIint.Bl, MF.103^{-}11b^{+}24^{-}, MF.Lu and MF.II^{-}480hi.PC, hybridised using Affymetrix Mouse Gene 1.0 ST array) and GSE49358 (P4 and P5 dermal macrophage subsets) were extracted and together processed similarly as processing GSE49358 in the comparative analysis of APC subsets between human and mouse described earlier. Monocyte and macrophage subsets as one group were compared with DC subsets as another group. For both human data and mouse data, the differentially expressed genes (DEGs) were identified using t-test with BH multiple testing corrected p-value of <0.05 and fold-change of >1.5. The NCBI HomoloGene table (build 67) was used to map the DEGs between human and mouse. Only DEGs with one-to-one ortholog mapping between the two species were kept. Pearson correlation analysis was done to compare the log fold-change values of the conserved human and mouse DEGs for monocytes and macrophages compared to DCs (*r=0.58* and *p-value = 2.2E-16)*.

All the data processing and analyses were carried out using the R programming language version 2.12.2 (http://www.r-project.org) and Pipeline Pilot (http://accelrys.com/products/pipeline-pilot/)

Antibodies used for flow cytometry and microscopy

The following antibodies were used for flow cytometry: CD45 v500 (H130, BD Horizon), HLA-DR percpcy5.5 (LN3, Ebioscience), HLA-DR PE (L243, ebioscience), CD14 APCCy7 (61D3, Ebioscience), CD1a A700 (HI149, Biolegend), CD1c Pecy7 (L161, Biolegend). CD11c v450 (B-ly6, BD Pharmingen), CD141 APC (AD5-14H12, Miltenyi), DC-SIGN PE (DCS-8CI, Biolegend), CD163 PE (215927, R&D), BTLA PE (MIH26, Biolegend), CD11b PE (ICRF44, Biolegend), SIRP1a PE (SESA5, Biolegend), CX3CR1 PE (2A9-1, Biolegend), CD26 PE (BA5b, Biolegend), CD64 PE (10.1, Biolegend), IRF4 PE (M-17, Sant Cruz), FLT3 PE (4G8, BD Pharmingen), MCSFR PE (12-3A3-1B10, Ebioscience). The following antibodies were used for microscopy: CD11c (B-ly6, BD Biosciences), CD11c FITC (BU15, AbD seotec) HLA-DR FITC (L243, BD Biosciences), LYVE1 (Polyclonal, R&D), FXIIIa (polyclonal, Enzyme Research Lab), DCSIGN (5D7, Abcam); donkey anti-rabbit and donkey anti-mouse Dy488, Dy549 or Dy649 (Jackson ImmunoResearch) and donkey anti-goat and donkey anti-sheep AlexaFluor647 or AlexaFluor488 (Invitrogen). Antibodies used for mouse analysis were purchased from BD, eBioscience and R&D: CD14

(Sa14-2), CD11b (M1/70), CD24 (M1/69), CD45 (30-F11), CD64 (X54-5/7.1), MHC2 (M5/114.15.2), CCR2 (475301) and Ly6C (HK1.4).

RQ-PCR primer details

Human Gene	Assay ID	RefSeq	Exon Boundary	Assay Location	Amplicon Length
GAPDH	4352934E	NM_002046.3	3-4	Not Given	122
LYVE1	Hs00272659_m1	NM_006691.3	1-2	373	68
CD209	Hs01588349_m1	NM_021155.3	6-7	1078	131
ZBTB46	Hs01008168_m1	NM_025224.3	4-5	1595	59
F13A1	Hs00173388_m1	NM_000129.3	2-3	254	80
GGT5	Hs00269779_m1	NM_001099781.1	11-12	2036	63
IL1A	Hs00174092_m1	NM_000575.3	6-7	1578	69

Figure S1, related to Figure 3

A and B. Ingenuity^{TM} Pathway Analysis (IPA) of the differentially expressed genes (DEGs) comparing monocytes, macrophages and CD14$^+$ cells (monocyte-macrophage group) to CD141$^+$ DCs and CD1c$^+$ DCs (DC group). DEGs were identified using t-test with BH multiple testing corrected for p-value <0.05 and fold change >1.5 between the two groups. Using these criteria, 301 up-regulated and 278 down-regulated genes (monocyte-macrophage group vs DC group) were identified. The graphs show enriched pathways for the up and down regulated genes. Blue bars indicate the p values (-log10) for pathway enrichment. The yellow squares indicate the ratio of the number of up or down-regulated genes mapped to the enriched pathway to the total number of molecules on that pathway represented by the dashed yellow line. The horizontal solid yellow line corresponds to the p-value = 0.05 threshold.

Figure S1

A Upregulated pathways in human monocytes and macrophages compared to DCs

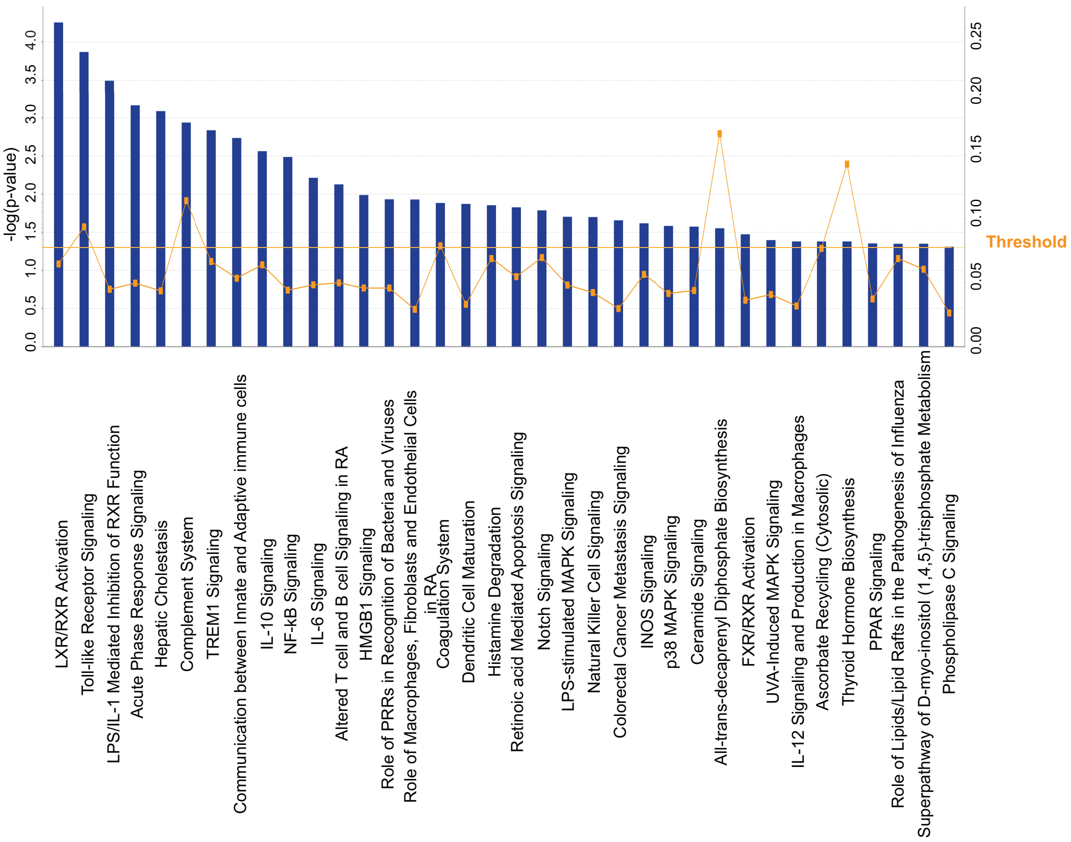

B Downregulated pathways in human monocytes and macrophages compared to DCs

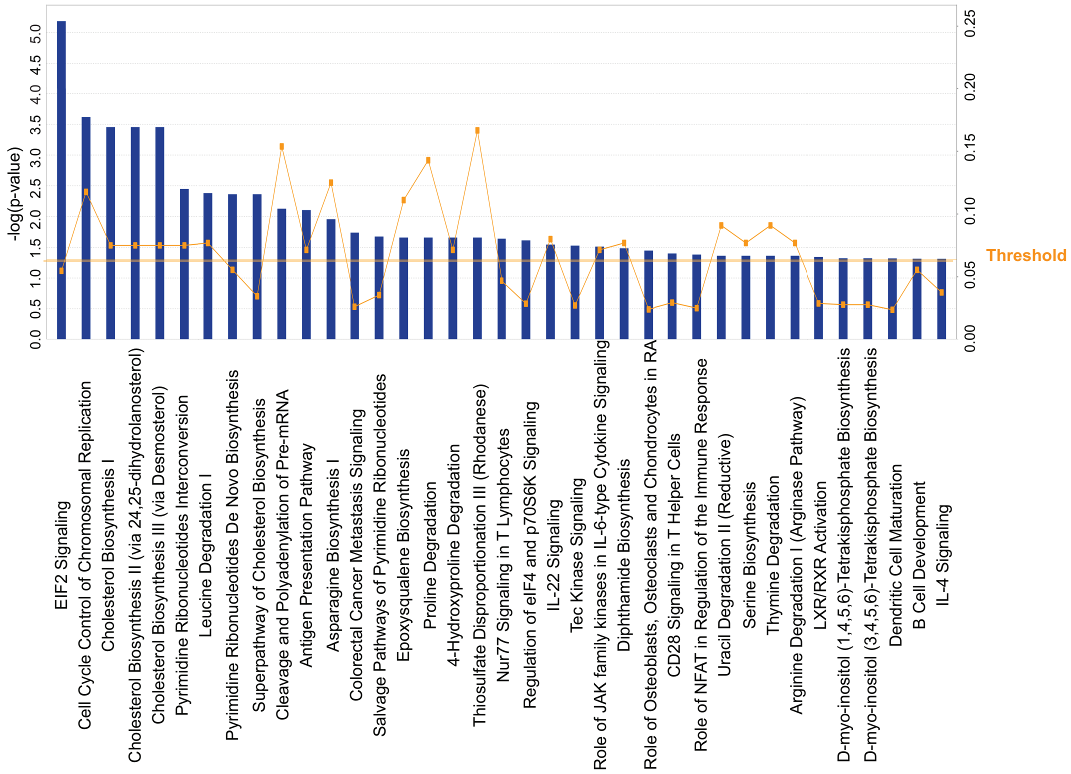

Figure S2, related to Figure 4

A. Overlay histogram showing DCSIGN expression analysed by flow cytometry by CD14$^+$ cells (blue) and CD1c+ DCs (green) spontaneously migrated from skin at 24 hr and 48 hr of skin explant culture. Isotype control shown in grey, representative data from n=4 independent donors shown.

B. DCSIGN expression evaluated by fluorescence microscopy analysis of cytospin prepared migrated cells from skin explant cultured for 60 hr. Representative image from n= 3 donors shown.

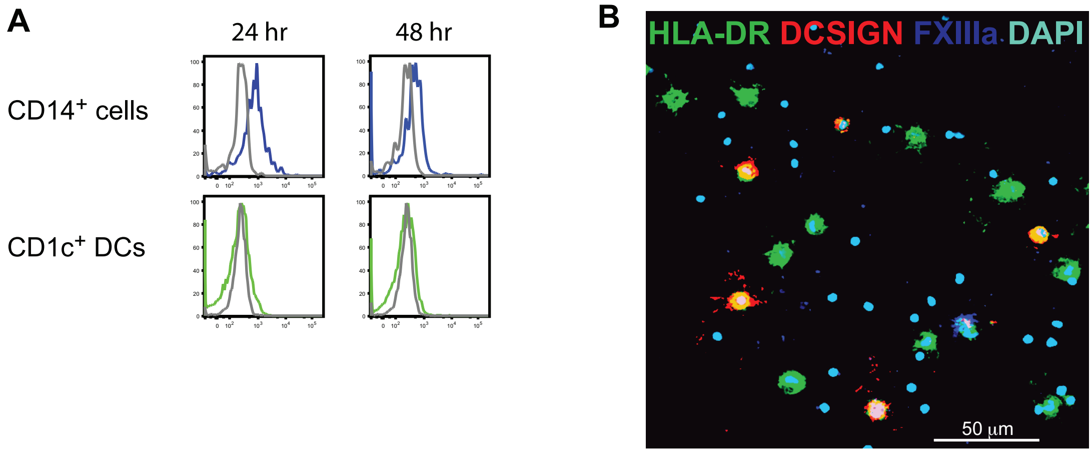

Table S1, related to Figure 2. Clinical data of HSCT patients

Patient	Diagnosis	Donor	Conditioning Regime	Alemtuzumab Dose
1	AML	MUD	FLAMSA	30
2	lymphoma	Sib	Flu Mel	30
3	AML	MUD	FLAMSA	30
4	AML	Sib	FLAMSA	30
5	MDS	MUD	FLAMSA	30
6	AML	MUD	Flu Bu	30
7	lymphoma	Sib	Flu Mel	30
8	lymphoma	MUD	Flu Mel	60
9	ALL	MUD	Flu Mel	60
10	AML	Sib	Flu Bu	30
11	MDS	MUD	Flu Bu	60
12	MDS	MUD	Flu Bu	60
13	lymphoma	MUD	Flu Mel	60
14	AML	MUD	Flu Bu	30
15	MDS	MUD	FLAMSA	30
16	lymphoma	MUD	Flu Mel	60
17	AML	MUD	Flu Bu	60

AML = acute myeloid leukaemia
MDS = myelodysplastic syndrome
MUD = matched unrelated donor
Sib = matched sibling donor
FLAMSA = fludarabine, amsacarine, ara-C
Flu Bu = fludarabine busulphan
Flu Mel = fludarabine melphalan
