# Supplementary material for: Human Dermal CD14+ Cells Are a Transient Population of Monocyte-Derived Macrophages
Source: Immunity. 2014 Sep 18;41(3):465–77. doi: 10.1016/j.immuni.2014.08.006 (PMC4175180; doi:10.1016/j.immuni.2014.08.006)
Supplement: Document S3. Article plus Supplemental Information [file mmc3.pdf]

CellPress

Human Dermal $CD14^+$ Cells Are a Transient Population of Monocyte-Derived Macrophages

Naomi McGovern,[1,2,4] Andreas Schlitzer,[2,4] Merry Gunawan,[1,4] Laura Jardine,[1] Amanda Shin,[2] Elizabeth Poyner,[1] Kile Green,[1] Rachel Dickinson,[1] Xiao-nong Wang,[1] Donovan Low,[2] Katie Best,[1] Samuel Covins,[1] Paul Milne,[1] Sarah Pagan,[1] Khadija Aljefri,[1] Martin Windebank,[1] Diego Miranda Saavedra,[1] Anis Larbi,[2] Pavandip Singh Wasan,[1] Kaibo Duan,[2] Michael Poidinger,[2,3] Venetia Bigley,[1] Florent Ginhoux,[2,5] Matthew Collin,[1,5,*] and Muzlifah Haniffa[1,5,*]
[1]Institute of Cellular Medicine, The Medical School, Newcastle University, Framlington Place, Newcastle upon Tyne NE2 4HH, UK
[2]Singapore Immunology Network, Agency for Science Technology and Research (A-Star), 8A Biomedical Grove, Immunos, Singapore 138648
[3]Department of Biological Sciences, National University of Singapore, 14 Science Drive 4, Singapore 117543
[4]Co-first author
[5]Co-senior author
*Correspondence: matthew.collin@newcastle.ac.uk (M.C.), m.a.haniffa@newcastle.ac.uk (M.H.)
http://dx.doi.org/10.1016/j.immuni.2014.08.006
This is an open access article under the CC BY license (http://creativecommons.org/licenses/by/3.0/).

SUMMARY

Dendritic cells (DCs), monocytes, and macrophages are leukocytes with critical roles in immunity and tolerance. The DC network is evolutionarily conserved; the homologs of human tissue $CD141^{hi}$ $XCR1^+CLEC9A^+$ DCs and $CD1c^+$ DCs are murine $CD103^+$ DCs and $CD64^-CD11b^+$ DCs. In addition, human tissues also contain $CD14^+$ cells, currently designated as DCs, with an as-yet unknown murine counterpart. Here we have demonstrated that human dermal $CD14^+$ cells are a tissue-resident population of monocyte-derived macrophages with a short half-life of <6 days. The decline and reconstitution kinetics of human blood $CD14^+$ monocytes and dermal $CD14^+$ cells in vivo supported their precursor-progeny relationship. The murine homologs of human dermal $CD14^+$ cells are $CD11b^+CD64^+$ monocyte-derived macrophages. Human and mouse monocytes and macrophages were defined by highly conserved gene transcripts, which were distinct from DCs. The demonstration of monocyte-derived macrophages in the steady state in human tissue supports a conserved organization of human and mouse mononuclear phagocyte system.

INTRODUCTION

Dendritic cells (DCs) and macrophages are a heterogeneous population of leukocytes that are critical in orchestrating immune responses (Steinman, 2007). Human tissues are populated by at least three DC subsets; $CD141^{hi}$ DCs (Haniffa et al., 2012; Watchmaker et al., 2014), $CD1c^+$ DCs (Lenz et al., 1993; Morelli et al., 2005; Angel et al., 2006; Zaba et al., 2007), and $CD14^+$ DCs (Nestle et al., 1993; de Gruijl et al., 2006; Klechevsky et al., 2008; Haniffa et al., 2009). Gene-expression studies suggest that human blood and tissue $CD141^{hi}$ DCs are homologous to murine tissue $CD103^+$ and splenic $CD8^+$ DCs (Robbins et al., 2008; Bachem et al., 2010; Crozat et al., 2010; Jongbloed et al., 2010; Poulin et al., 2010) and $CD1c^+$ DCs are homologous to $CD11b^+CD4^+$ DCs in the spleen and $CD11b^+CD24^+CD64^-$ DCs in nonlymphoid tissues (Schlitzer et al., 2013). However, the precise relationship of human $CD14^+$ DCs to murine tissue populations remains unclear (Haniffa et al., 2012). Excluding Langerhans cells of the epidermis, the apparent paradox of three DC subsets in human interstitial tissues but only two in murine tissues remains unreconciled.

Human $CD14^+$ DCs were first identified as a spontaneously migrating population from dermal explants cultured ex vivo. These cells were classified as DCs based on major histocompatibility complex (MHC) class II glycoprotein expression and their ex vivo migratory behavior. In vitro generated $CD14^+$ DCs from $CD34^+$ hematopoietic stem cells (HSCs) have been used alongside primary cells to dissect their immunological properties (Caux et al., 1996; Klechevsky et al., 2008; Morelli et al., 2005; de Gruijl et al., 2006; Angel et al., 2006; Haniffa et al., 2009; Haniffa et al., 2012; Matthews et al., 2012; Penel-Sotirakis et al., 2012). $CD14^+$ DCs secrete interleukin-10 (IL-10) and IL-6 and have been shown to induce regulatory T cells (Tregs) and helper follicular T cells (Tfh) (Chu et al., 2012; Klechevsky et al., 2008). A notable feature of $CD14^+$ DCs is their poor ability to stimulate allogeneic T cell proliferation (Klechevsky et al., 2008; Morelli et al., 2005; de Gruijl et al., 2006).

$CD14^+$ DCs also express CD141, which is further upregulated during spontaneous migration from skin explant culture and initially presumed to be related to blood $CD141^+$ DCs (Chu et al., 2012). More recently, the true counterpart of blood $CD141^+$ DCs has been shown to be tissue $CD14^-CD141^{hi}$ DCs (Haniffa et al., 2012). $CD14^+$ cells are related to human and mouse blood monocytes by gene expression and are rapidly reconstituted by donor-derived cells following hematopoietic stem cell transplantation (HSCT), unlike dermal macrophages, which turn over at a much slower rate (Haniffa et al., 2009; Haniffa et al., 2012).

In mice, steady-state DCs are derived from a lineage dependent on FLT3, in contrast to monocytes and macrophages, which are dependent on colony-stimulating factor-1 receptor (CSF-1R) (Yoshida et al., 1990; McKenna et al., 2000; Dai et al., 2002). Circulating murine $Ly6C^{hi}$ monocytes have been shown to extravasate into tissues existing as tissue monocytes

CrossMark

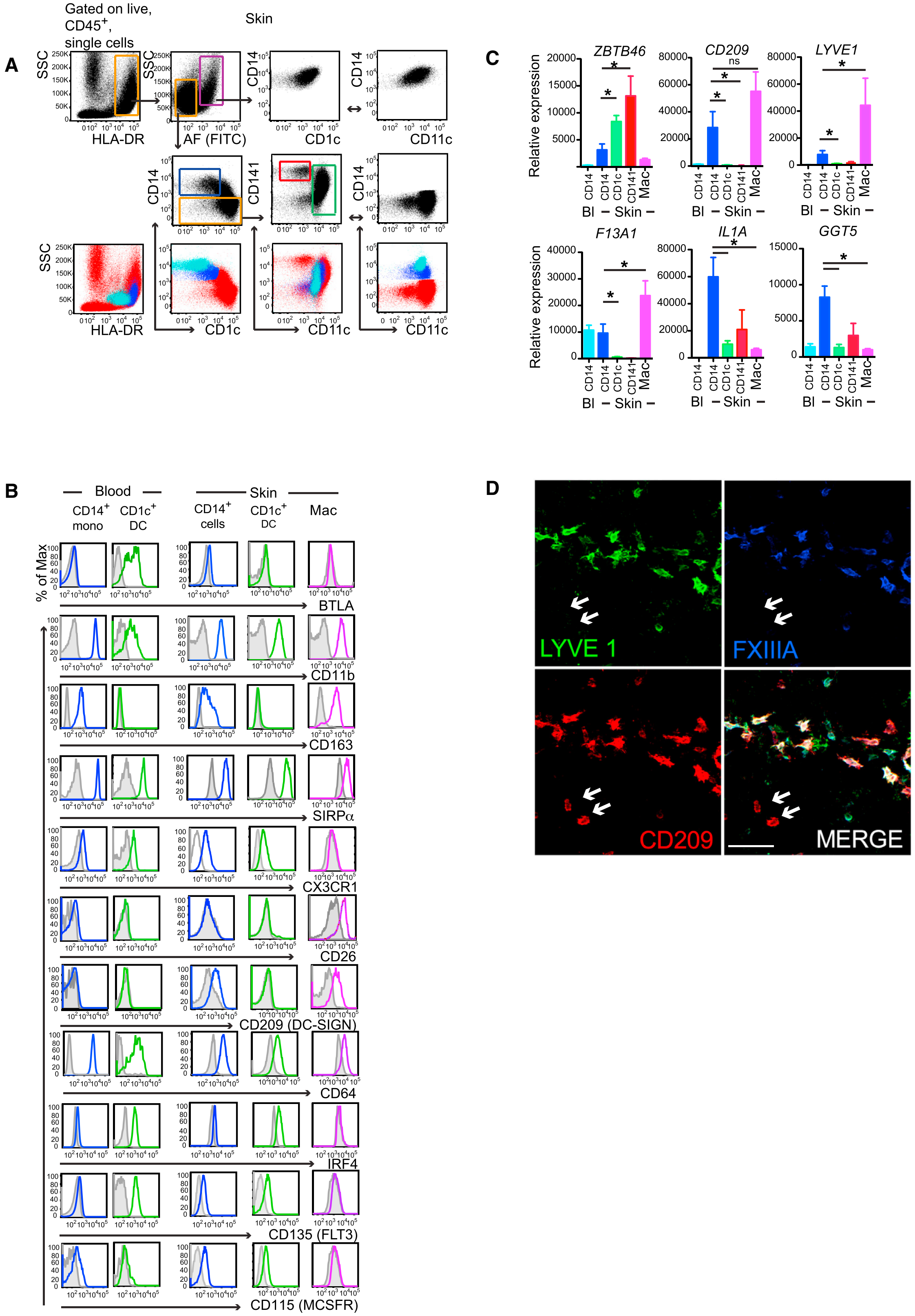

Figure 1. Tissue CD14⁺ Cells Are Phenotypically Related to Blood Monocytes and Tissue Macrophages

(A) Flow cytometry of enzymatically digested skin. Gating strategy used to identify tissue macrophages (AF⁺, purple gate), CD14⁺cells (blue gate), CD141⁺DCs (red gate), and CD1c⁺ DCs (green gate) is shown. (A, lower panel) Overlay dot plots of CFSE-labeled purified blood CD14⁺ monocytes (cyan) cultured with

(legend continued on next page)

(Jakubzick et al., 2013; Tamoutounour et al., 2012) and also differentiate into DC-like and macrophage populations in the intestine and dermis (Bogunovic et al., 2009; Varol et al., 2009; Tamoutounour et al., 2012; Yona et al., 2013). Monocytes as a source of tissue inflammatory DCs are also well-documented (Zigmond et al., 2012; Plantinga et al., 2013; Tamoutounour et al., 2013). Human blood monocyte differentiation into DCs has been proposed in inflammation as the potential equivalent of in vitro cultured GM-CSF and IL-4 monocyte-derived DCs (Segura et al., 2013). However, the precise contribution of circulating monocytes to human tissue DCs and macrophages in steady state is unclear.

Altogether, these findings led us to question whether CD14^{+} cells were bona fide DCs and which murine population was their homolog. In this study, we investigated the relationships between circulating blood CD14^{+} monocytes and tissue macrophages with tissue MHC classII^{+}CD14^{+} cells, currently defined as "DCs." We defined the transcriptomic profile of the human monocyte-macrophage lineage distinct from the DC lineage and demonstrated the conserved gene transcripts defining these two lineages in humans and mice. Our findings revealed that CD14^{+} cells more closely resemble tissue resident, monocyte-derived macrophages than bone fide DCs. In addition, we showed that the murine dermal monocyte-derived macrophages are the homolog of human dermal CD14^{+} cells.

RESULTS

Tissue CD14^{+} Cells Are Phenotypically Related to Blood Monocytes and Interstitial Macrophages

We previously showed that CD14^{+} cells were distinct from dermal macrophages, which possessed dense cytoplasmic melanin granules by morphology, were autofluorescent by flow cytometry analysis, were adherent and nonmigratory, although both populations express CD14 (Haniffa et al., 2009). As the existence of tissue monocytes derived from Ly6C^{hi} classical monocytes was recently demonstrated in mice (Jakubzick et al., 2013; Tamoutounour et al., 2013), we therefore first set out to determine the phenotypic distinctions between dermal MHC classII^{hi} autofluorescent (AF)$^{-}$CD14^{+} cells (hereafter referred to as CD14^{+} cells), AF^{+}CD14^{+} macrophages (hereafter referred to as dermal macrophages), and blood CD14^{+} monocytes, the homologs of murine Ly6C^{hi} monocytes. To control for the preparation conditions of freshly isolated CD14^{+} cells by enzymatic digestion, we cultured CFSE-labeled purified blood CD14^{+} monocytes with enzymatically-digested skin overnight. Gating on CFSE-labeled cells allowed direct comparison of tissue CD14^{+} with CD14^{+} monocytes. Overlay dot plot of CFSE-labeled CD14^{+} monocytes on digested skin cells showed that skin CD14^{+} cells were phenotypically distinct from blood monocytes with higher side scatter (SSC) properties, expressing higher amounts of HLA-DR and CD1c (Figure 1A). Both skin CD14^{+} cells and CFSE-labeled CD14^{+} monocytes spiked into the skin preparation had variable expression of CD141 (Figure 1A). We noted very few CD14^{+} cells with an identical profile to the CFSE-labeled monocytes cultured with digesting skin (Figure 1A), which might represent extravasated tissue monocytes in healthy human skin.

We next compared the expression of selected antigens characterizing monocyte-macrophage cells in human skin and blood-antigen-presenting cell populations (Figure 1B). Unlike CD1c^{+} DCs from blood and skin, CD14^{+} cells had variable expression of CD163 similar to blood monocytes and macrophages (Figure 1B). We next compared the expression of skin antigen-presenting cell subsets and blood CD14^{+} monocytes for the following transcripts; DC transcription factor (TF) *ZBTB46*, *CD209*, lymphatic vessel endothelial hyaluronan receptor (*LYVE1*), and factor XIIIa (*F13A1*). The latter three antigens were shown to identify dermal macrophages in situ (Wang et al., 2014) (Figure 1C). Skin CD1c^{+} and CD141^{hi} DCs expressed higher amounts of *ZBTB46* transcripts and low amounts of *CD209* and *LYVE1*. In contrast, dermal CD14^{+} cells expressed 40% and macrophages 15% of *ZBTB46* transcript amounts compared to CD1c^{+} DCs, consistent with previous reports on human inflammatory DCs and murine dermal macrophage populations (Segura et al., 2013; Tamoutounour et al., 2013). CD14^{+} cells and macrophages expressed higher amounts of *CD209* transcript compared to all other subsets, but *LYVE1* transcript expression was highest in macrophages (Figure 1C) similar to *F13A1* expression (Haniffa et al., 2009). In addition, dermal CD14^{+} cells expressed high amounts of *IL1A* and gamma-glutamyl transferase 5 (*GGT5*) transcripts (Figure 1C), which were identified from our previous microarray analysis (Haniffa et al., 2012). Immunostaining of whole-mount dermal sheet for LYVE-1, CD209 (DC-SIGN), and FXIIIa identified LYVE-1^{-}FXIIIa^{lo}DC-SIGN^{+} cells corresponding to the CD14^{+} cells in situ (Figure 1D).

Skin CD14^{+} Cells Are Derived from CD14^{+} Blood Monocytes

Human tissue DCs are depleted in patients lacking circulating DCs and monocytes as shown by patients with genetic deficiency of monocytes and DCs due to *GATA2* or *IRF8* mutation, but the exact precursor-progeny relationships are difficult to demonstrate conclusively in humans (Bigley et al., 2011; Hambleton et al., 2011). We previously showed that skin CD14^{+} cells are derived from donor bone marrow within 40 days of HSCT (Haniffa et al., 2009), but the kinetics of this relationship have not been resolved in detail. In this study, we followed the course of blood and skin monocytes, macrophages, and DCs during preparative cytotoxic therapy for transplantation and for up to 2 weeks after HSCT (clinical data in Table S1).

enzymatically digested skin (red) and phenotypically compared to CD14^{+} cells (blue). Bidirectional arrows depict equivalent cells. Corresponding plots in middle panel and SSC versus HLA-DR from top panel is shown as red. Representative data from at least four skin donors are shown.

(B) Relative expression of selected antigens on blood CD14^{+} monocytes and CD1c^{+} DCs, skin CD14^{+} cells, CD1c^{+} DCs, and macrophages compared to isotype control (gray). Representative data from at least three donors are shown.

(C) Relative expression of *ZBTB46*, *DCSIGN*, *LYVE1*, *F13A1*, *IL1A*, *GGT5* mRNA by skin CD14^{+} cells, CD1c^{+} DC, CD141^{+} DC, macrophages, and blood CD14^{+} monocytes. Composite data from six donors is shown, mean ± SEM, *$p < 0.05$, Mann-Whitney U test.

(D) Pseudocolor images of whole-mount skin immunostained for LYVE-1 (green), CD209 (DCSIGN) (red), and FXIIIa (blue). White arrows identify LYVE-1^{-}, DC-SIGN^{+}, FXIIIa$^{-/lo}$ cells corresponding to CD14^{+} cells. Scale bar represents 50 μm. Representative image from at least four donors is shown.

Cytotoxic therapy induced bone-marrow suppression and absolute monocytopenia by the day of transplantation (day 0) (Figure 2A). This was mirrored by a rapid loss of CD14$^+$ cells from the skin within 6 days. After a delay of 6 days, macrophage numbers also declined to approximately 50%, where they remained stable. In the early recovery phase after HSCT, there was a rapid rise in CD14$^+$ blood monocytes, which coincided with a rapid reconstitution of skin CD14$^+$ cells (Figure 2B). The kinetics of DC recovery were slower in the blood and skin and did not attain the same frequency as healthy controls (Figures 2A and 2B). The temporal relationship between blood CD14$^+$ monocytes and skin CD14$^+$ cells is consistent with a precursor-progeny relationship in which CD14$^+$ monocytes give rise to CD14$^+$ cells within a short timeframe. In support of the rapid differentiation step of CD14$^+$ monocytes into skin CD14$^+$ cells suggested by our in vivo findings, we showed that purified CD14$^+$ monocytes upregulated antigens and adopted morphological changes characteristic of CD14$^+$ tissue cells upon culture with primary endothelial cells over 3 days (Figure 2C).

Skin CD14$^+$ Cells Are Transcriptionally Aligned to Human Monocytes and Macrophages

In order to evaluate the lineage identity of human skin CD14$^+$ cells, we performed microarray transcription profiling of human skin and blood dendritic cells, macrophages, and monocyte subsets. Principal component analysis (PCA) of all subsets analyzed showed separation of DCs from monocyte-macrophages in component one and further definition between monocyte-macrophage subsets and plasmacytoid DCs (pDCs) from myeloid DCs in component 2 (Figure 3A). We also performed a supervised connectivity map (CMAP) gene set enrichment analysis and showed that the CD14$^+$ cell gene set was enriched in dermal macrophages and also weakly in CD14$^+$ blood monocytes but exhibited an inverse expression profile to blood and skin DCs (Figure 3B). This suggests that CD14$^+$ cells are more closely aligned to tissue macrophages and CD14$^+$ blood monocytes than to blood or tissue DCs.

Further examination of the relationship between CD14$^+$ cells, monocytes, macrophages, and DCs revealed a number of coregulated genes distinguishing monocytes, CD14$^+$ cells, and macrophages from blood and skin DCs (Figure 3C). Functional pathways identified by the monocyte, CD14$^+$ cell, and macrophage gene sets include retinoid X receptor signaling, TREM1 signaling, complement system, and communication between innate and adaptive cell regulation (see Figure S1 available online). In contrast, the human DC gene signature was enriched for cell-cycle control and amino acid, nucleic acid and cholesterol metabolism pathways (Figure S1).

We next performed cross-species analysis comparing human monocyte, macrophage, and CD14$^+$ cells with murine monocytes, macrophages, and DC subsets obtained from ImmGen (Gautier et al., 2012) and GSE49358 (Tamoutounour et al., 2013) microarray data sets. This analysis identified a set of genes that are differentially expressed in a conserved manner, which include *SLC11A1*, *MAFB*, *CD14*, and *FCGR2A* (Figure 3D; Table S2). Similarly, human and mouse DC lineage also shared close homology of transcripts across species including *FLT3*, *BTLA*, *HLA-DOA*, and *CIITA* (Figure 3D; Table S2).

Spontaneous Migration of Skin CD14$^+$ Cells Does Not Occur via Lymphatic Vessels

A defining property of tissue-resident DCs is their migratory capacity to lymph node (LN) directed by CCL19 and CCL21 signaling through CCR7. In vitro culture of explanted tissue mimics this process and resident DCs might be observed entering the lymphatic channels prior to emigrating from the tissue (Stoitzner et al., 1999; Ohl et al., 2004; Wang et al., 2014). In addition, this experiment showed that resident macrophages remain fixed in the tissue (Haniffa et al., 2009; Wang et al., 2014). The ability of CD14$^+$ cells to leave tissue explants has been invoked as a DC credential (Nestle et al., 1993), but their route of migration in the ex vivo assay has not yet been established. If CD14$^+$ cells are not fixed in skin explants, then simple redistribution in ex vivo culture would result in apparent emigration from the tissue. Like dermal macrophages, migrated and digested CD14$^+$ cells did not express CCR7 even upon stimulation (Figure 4A) (Haniffa et al., 2009). Time-course analysis of skin explants showed the presence of CD14$^+$ cells in the skin explant medium as early as 12 hr after culture (Figure 4B). However, at no stage were CD14$^+$ cells observed within lymphatics as assessed by whole-mount immunostaining of skin explants (Figure 4C). DC-SIGN expression is retained by spontaneously migrating CD14$^+$ cells (Figure S2) and would have permitted their localization within lymphatic channels if this had been the route of migration. The presence of spontaneously migrated CD14$^+$ cells, despite their absence within skin lymphatic lumen, suggests that CD14$^+$ cells exited from the skin without entering the lymphatic vasculature. Dermal macrophages did not migrate spontaneously (Figure 4C), in keeping with previous observations (Haniffa et al., 2009).

Migratory CD14$^+$ cells have been much studied, but their relationship to CD14$^+$ cells isolated by enzymatic digestion has not been extensively evaluated. We compared cells isolated by digestion and migration for the expression of a custom selection of 96 genes reported in the literature to define DCs and macrophages by Taqman Low Density Array PCR. Unsupervised clustering showed that migrated and digested CD1c$^+$ DCs and CD14$^+$ cells clustered by subset rather than isolation method (Figure 4D). *CCR7*, *CYP27B1*, *FLT3*, *FSCN1*, *INDO*, *LAMP3*, and *LY75* were expressed at higher amounts by CD1c$^+$ DCs compared to CD14$^+$ cells, which expressed higher amounts of *CCL18*, *CCL3*, *CCR1*, *CD163*, *CD36*, *CLEC10A*, *FCGR1A* and *3A*, *IL10*, *MARCO*, *MMP12*, *MSR1*, *SIGLEC1*, and *TREM2* (Figure 4D), transcripts characteristic of monocyte-macrophage cells.

Skin CD14$^+$ Cells Are Potent Inducers of Memory T Cell Response but Poor Stimulators of Naive T Cells

A cardinal property of DCs as opposed to monocytes and macrophages is their superior ability to activate naive T cell proliferation. It has been previously shown that CD14$^+$ cells from the skin are inferior to CD1c$^+$ DCs in inducing allogeneic naive T cell proliferation (Klechevsky et al., 2008; Angel et al., 2006), but a direct comparison with dermal macrophages has never been performed. After 5 days culture with CFSE-labeled allogeneic naive CD4$^+$ T cells, CD14$^+$ cells induced 80% lower proliferation of naive CD4$^+$ T cell compared to CD1c$^+$ DCs with negligible T cells proliferation observed with macrophages

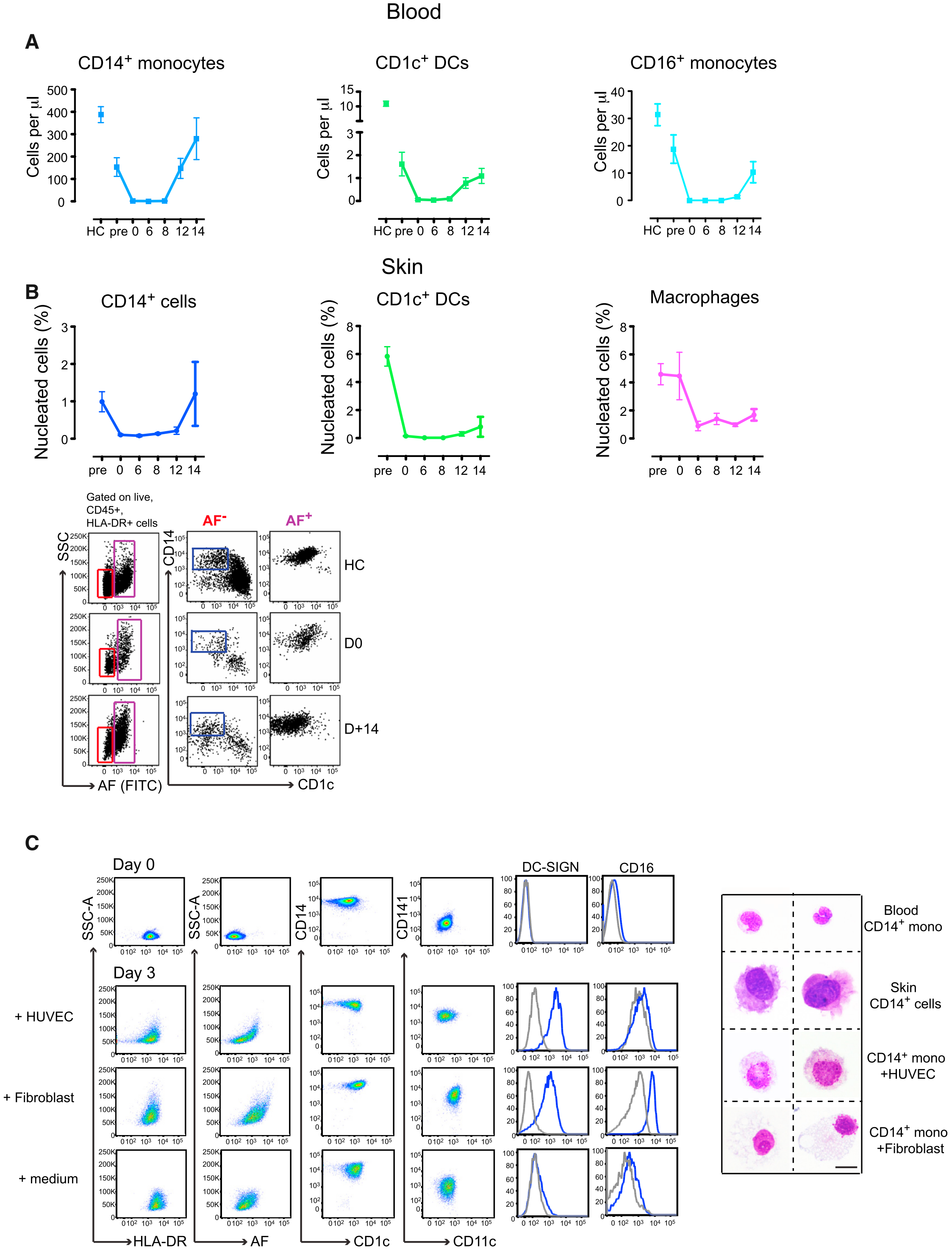

Figure 2. Skin CD14⁺ Cells Are Derived from CD14⁺ Blood Monocytes

(A) Absolute count of blood CD14⁺ and CD16⁺ monocyte subsets and CD1c⁺ DCs upon conditioning and up to 14 days after HSCT. HC, healthy controls. Data from 17 patients and 15–20 HC are shown, mean ± SEM.

(B) Frequency of skin CD14⁺ cells, CD1c⁺ DCs and macrophages upon conditioning and up to 14 days after HSCT as a % of nucleated cells. Data from 17 patients are shown. Mean ± SEM. A maximum of two skin biopsies per patient were taken at different time points and were collagenase digested. Bottom panel depicts representative dot plots of skin flow-cytometry analysis.

(C) Phenotype and morphology of CD14⁺ blood monocytes after culture with medium alone, HUVECS or fibroblasts for 0–3 days. Scale bar represents 10 µm. Representative data from nine different donors is shown. Overlay histogram of DC-SIGN and CD16 expression (blue) compared to isotype control (gray).

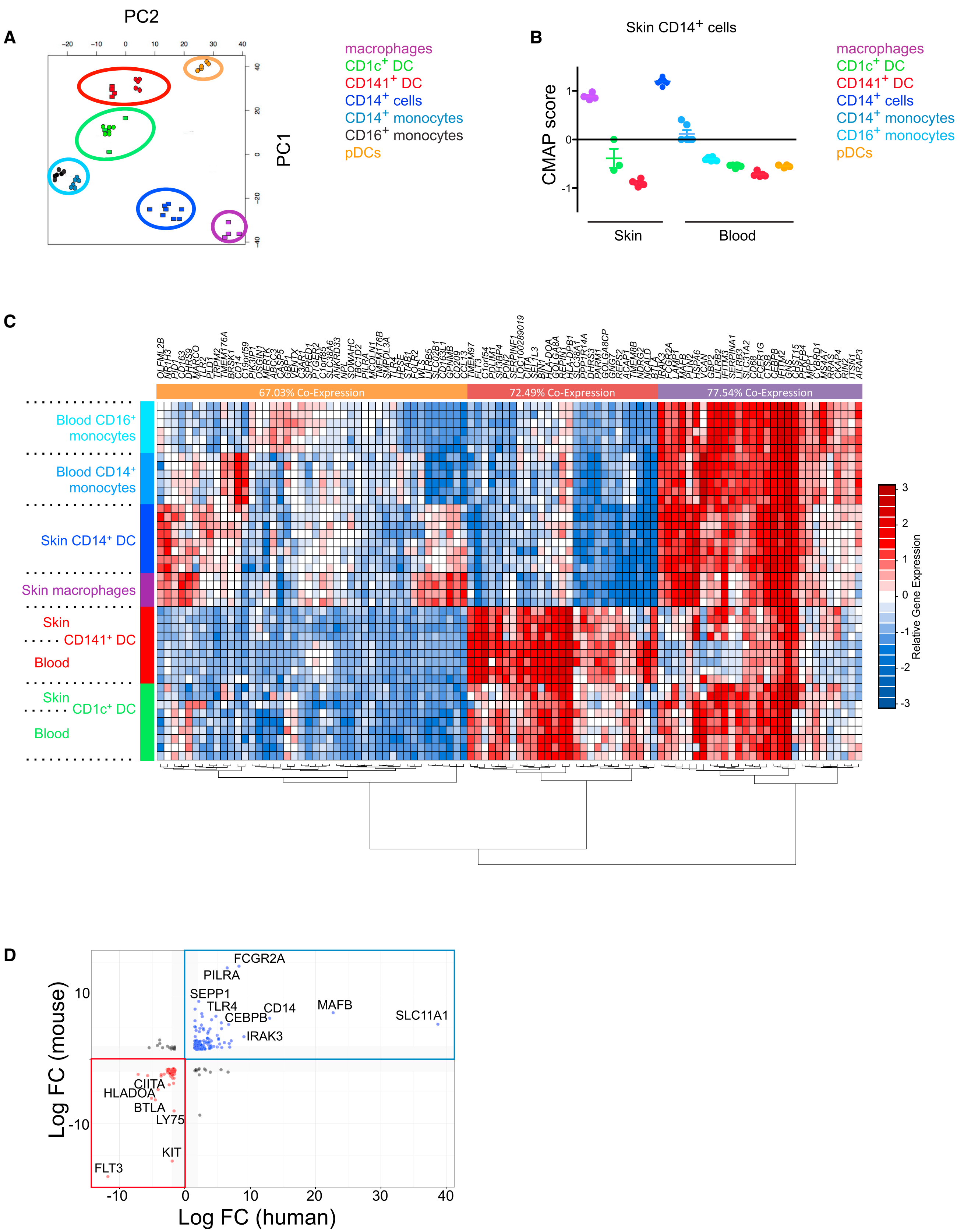

(legend on next page)

even at the highest APC: T cell ratio tested of 1:10 (Figure 5A). However, CD1c$^+$ DCs, CD14$^+$ cells, and dermal macrophages were potent inducers of memory CD4$^+$ T cell proliferation and cytokine production upon stimulation with *Candida albicans* (Figure 5B). Both CD14$^+$ cells and dermal macrophages were comparable to CD1c in their ability to induce IL-17, IL-22, interferon-γ (IFN-γ), and IL-4 production by memory CD4$^+$ T cells (Figure 5B).

Murine CD11b$^+$Ly6C$^-$CD64^{lo-hi} Monocyte-Derived Macrophages Are the Homolog of Human CD14$^+$ Cells

Recent studies have demonstrated heterogeneity within murine nonlymphoid tissues CD11b$^+$ cells, which comprise DCs, monocytes, monocyte-derived DCs, and resident macrophages (Langlet et al., 2012; Tamoutounour et al., 2012). In order to precisely identify the murine equivalent of human CD14$^+$ cells, we performed comparative transcriptomics analysis by using microarray data of the recently described CD11b$^+$ monocyte, DC, and macrophage populations in murine dermis (Tamoutounour et al., 2013). CMAP analysis revealed highest enrichment of human CD1c$^+$ DCs to their murine CD11b$^+$ DC counterpart in both steady state and upon inflammation induced by the contact hypersensitivity allergen DNFB (Figure 6A). The reciprocal relationship was also observed that human dermal macrophages had low but positive CMAP enrichment scores with murine macrophages and monocyte-derived cells (Figure 6A). Blood CD14$^+$ monocytes had the highest enrichment score with mouse Ly6C^{hi} blood monocytes in the steady state (Figure 6A), in keeping with previous analysis (Haniffa et al., 2012). Human CD14$^+$ cells were most enriched with murine macrophages (P4 and P5) followed by Ly6C^{lo}MHC class II$^+$ monocyte-derived DC-like cells (P2 and P3) (Figure 6A). These results suggest that human CD14$^+$ cells are related to monocytes but are not the equivalent of murine tissue monocytes (P1). The existence of murine monocyte-derived dermal macrophages was recently reported by Tamatounour et al. as observed by a reduction in P4 and P5 dermal macrophages in *Ccr2*$^{-/-}$ mice. We therefore hypothesized that human CD14$^+$ cells were the homolog of murine dermal monocyte-derived macrophages. In order to confirm the monocyte origin of some murine dermal macrophages, we used the *S100a4-cre* × *R26*^{YFP}mice in which >99% of hematopoietic stem cells (HSCs) and resultant blood monocytes express yellow fluorescent protein (YFP). This fate-mapping model previously demonstrated the independence of resident tissue macrophages from circulating monocytes and HSC progenitors (Hashimoto et al., 2013). Our analysis of mouse dermal DC and macrophage fractions showed that >90% of CD11b$^+$ P1-P3 and 80%–90% of P4 and P5 macrophages were indeed monocyte-derived (Figure 6B). Collectively, these results provide further evidence of functional equivalence between monocyte-derived macrophages in the mouse dermis as the homologs of human CD14$^+$ cells.

DISCUSSION

The results presented here suggest that CD14$^+$ "DCs" are not related to the human DC lineage but are monocyte-derived macrophages that are resident in healthy skin. Their gene-expression program strongly overlaps with that of blood monocytes and resident tissue macrophages, but they are distinguishable from both of these by phenotypic and functional properties. Although it is difficult to prove unequivocally that they have a monocyte origin, their absence in monocyte deficiency states, kinetics of renewal after HSCT, and similarity to monocytes in short-term culture with endothelial cells are all consistent with a precursor-progeny relationship. Their evident lack of ability to stimulate naive T cell proliferation is consistent with their status as a monocyte-derived macrophage.

Tissue monocytes coexpressing Ly6C and MHC class II were recently demonstrated as a distinct population in murine tissues (Jakubzick et al., 2013; Tamoutounour et al., 2013). These cells, derived from Ly6C^{hi}MHC class II$^-$ circulating monocytes, upregulate MHC class II upon contact with endothelium (Jakubzick et al., 2013). In human, all blood and skin DCs, monocytes and macrophages express MHC class II (reviewed in Haniffa et al., [2013]). The Lin$^-$MHC class II$^-$ compartment in human blood does not contain any CD14-expressing cells and primarily comprises basophils (Autissier et al., 2010). "Spiking" CFSE-labeled blood CD14$^+$ monocytes into digesting skin allowed us to perform a direct comparison between dermal CD14$^+$ and blood monocytes.

The identity of the circulating precursors of human tissue DCs and macrophages has been a subject of intense debate. Despite the widespread use of in vitro culture protocols to generate monocyte-derived DCs and macrophages, in vivo evidence to support the contribution of such monocyte-derived cells in healthy tissue is limited (Chu et al., 2012). Here, we show the rapid decline and reconstitution of dermal CD14$^+$ cells that mirrors the kinetics of blood CD14$^+$ monocytes in patients undergoing HSCT. In contrast, blood and skin CD1c$^+$ DCs remained suppressed up to 14 days after HSCT and are thus unlikely to be the precursors of dermal CD14$^+$ cells. We also show that blood CD14$^+$ monocytes acquire the morphology and phenotypic characteristics of dermal CD14$^+$ cells upon culture with endothelial cells in line with previous reports (Randolph et al., 1998; Chomarat et al., 2000). The reduction of dermal macrophages after HSCT by approximately 50% is in keeping with the reduction in dermal macrophages in patients with *GATA2* and *IRF8* mutation who are deficient in peripheral blood DCs

Figure 3. Skin CD14$^+$ Cells Are Transcriptionally Aligned to Human Monocytes and Macrophages

(A) Principal component analysis of CD141$^+$ DCs, CD1c$^+$ DCs, CD14$^+$ cells, monocyte subsets, macrophages, and pDCs. Each symbol represents an individual sample. Rectangles depict skin subset and circles depict blood subset. Data from three to eight independent blood and skin donors are shown.

(B) CMAP enrichment scores for skin CD14$^+$ cells signature compared to human skin and blood monocytes and DC subsets. Each symbol represents an individual sample. Enrichment scores were significant at $p < 0.001$ for CD14$^+$ cell signature compared to other subsets.

(C) Heatmap showing 106 genes which were 2-log fold up (red) or downregulated (blue) in human monocyte-macrophages compared to DCs. $p < 0.001$ for each gene. Each row represents one sample.

(D) Scatterplot comparing genes that were >1.5 log fold up or downregulated in both human and mouse monocyte-macrophages (blue dots and square) compared to DCs (red dots and square). $p < 0.001$ for each transcript.

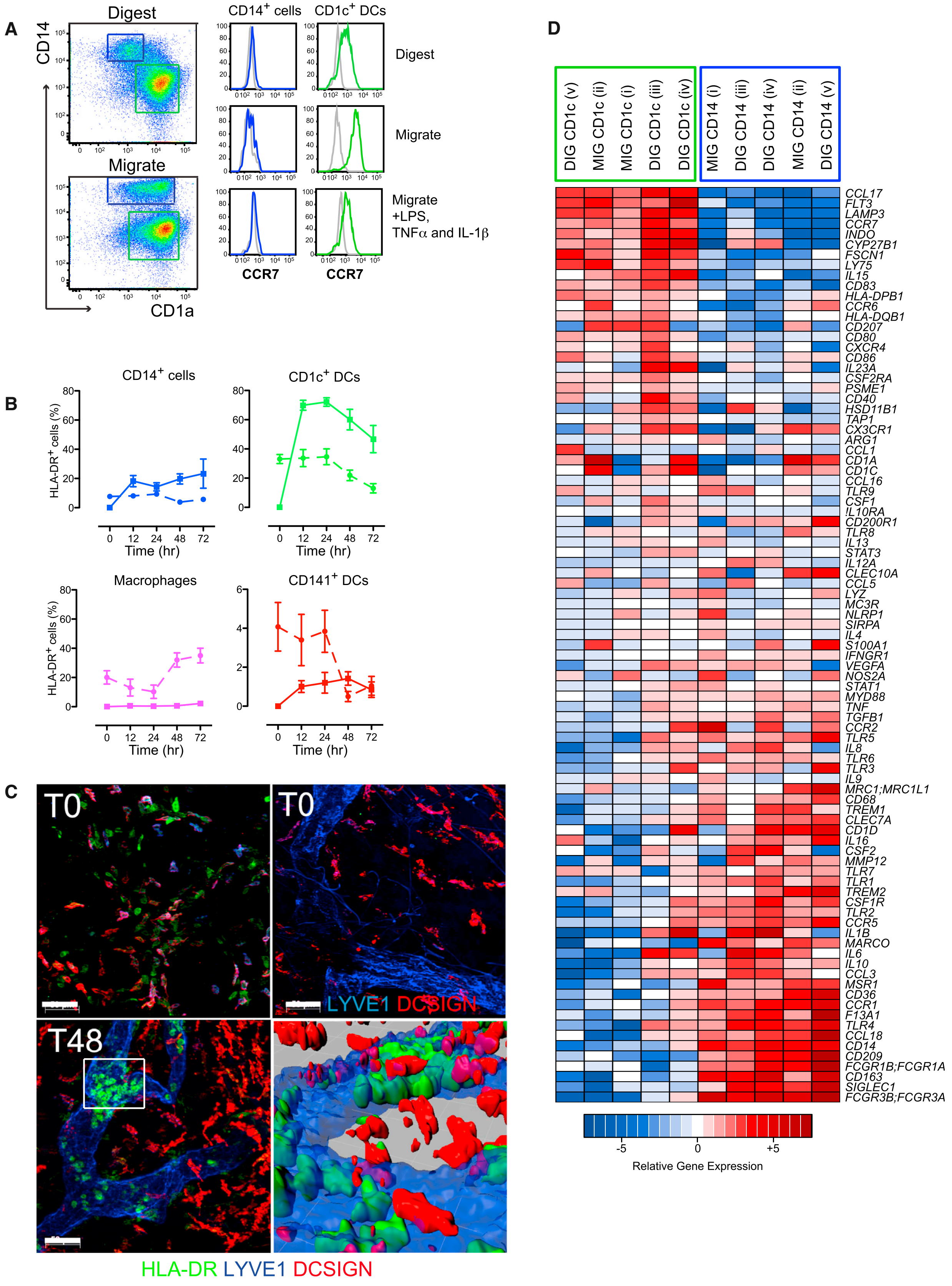

(legend on next page)

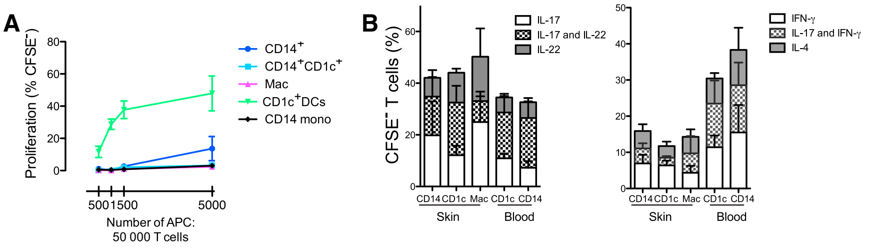

Figure 5. CD14$^+$ Cells Are Potent Inducers of Memory T Cell Response

(A) Naive T cell stimulation: proliferation of allogeneic naive CD4$^+$ T cells, (determined by CFSE dilution) after coculture for 6 days with CD14$^+$ cells, CD14$^+$CD1c$^+$ cells, macrophages, CD1c$^+$ DCs from skin and CD14$^+$ monocytes from blood (n = 5, mean ± SEM).

(B) Memory T cell stimulation: Intracellular expression of IL-17, IL-22, IL-4, and IFN-γ by PMA and ionomycin restimulated autologous CFSE-labeled bulk CD4$^+$ T cells following coculture with skin DC and macrophage subsets pulsed with *Candida albicans* (n = 6, mean ± SEM).

and monocytes (Bigley et al., 2011; Hambleton et al., 2011). Longer-term follow up after HSCT is required to evaluate macrophage recovery and whether proliferation of residual dermal macrophages or differentiation from CD14$^+$ cells is responsible for dermal macrophage reconstitution.

The utility of transcriptomics analysis to map homologous subsets between humans and mice (Robbins et al., 2008; Haniffa et al., 2012; Schlitzer et al., 2013; Watchmaker et al., 2014) and more recently to define DCs (Miller et al., 2012) and macrophages (Gautier et al., 2012) in mouse tissues is evident. Extending our previous CMAP analysis to human microarray data set incorporating dermal macrophages and epidermal LCs, we were able to precisely identify the monocyte-macrophage lineage of dermal CD14$^+$ cells. This identification enabled us to define the human DC and monocyte-macrophage transcriptomic signatures and to perform cross-species analysis with the murine DC, monocyte, and macrophage ImmGen data set. The extension of human DC, monocyte, and macrophage mapping further enhances the utility of the ImmGen data repository. Our analysis confirms a high-degree of conservation for DC and macrophage transcription networks between humans and mice identifying shared genes such as *MERTK*, *CD14*, and *SLC11A1* (*NRAMP*) to define monocytes and macrophage and *FLT3*, *BTLA*, and *KIT* to define DCs. Comparative biology analysis of DC subsets has supported the relevance of murine models for both developmental and functional studies and the extension of a similar analysis to the macrophage lineage could present an additional perspective for future studies on these cells.

The phenotypic and transcriptional assignment of dermal CD14$^+$ cells as monocyte-derived macrophages led us to reevaluate their ability to migrate spontaneously from skin explant cultures ex vivo which has been presumed to simulate lymphatic migration despite absent or very low expression of CCR7 even upon cytokine and LPS stimulation. We observed that although 1 in 5 HLA-DR$^+$ cells after 12 hr and 48 hr culture of skin explant were CD14$^+$ cells, these cells were not observed within skin lymphatic lumen. The dichotomy between spontaneous migration and lymphatic migration was previously observed with murine LCs and DCs from *Ccr7*$^{-/-}$ mice, which were capable of spontaneously migrating from skin explants but failed to form dermal cords indicative of lymphatic migration or enter skin draining lymph node (Ohl et al., 2004).

It is well documented that CD14$^+$ cells are poor stimulators of naive T cells, a property that is expected of tissue-resident cells. However, CD14$^+$ cells and macrophages are on par with DCs in regulating memory CD4$^+$ T cell responses as shown here and in previous reports. Dermal CD14$^+$ cells express high amounts of IL-1α and GGT5, a property not shared by any blood or skin DCs, monocytes, and macrophages, suggesting a role in maintaining epithelial integrity and regulation of skin inflammation including neutrophil migration (Han et al., 2002; Chen et al., 2007).

Murine tissue macrophages and epidermal Langerhans cells (LCs) were recently shown to arise from embryonic yolk sac and fetal liver precursors challenging the traditional dogma of monocytes as precursors of all tissue macrophages (van Furth and Cohn, 1968; Schulz et al., 2012; Hoeffel et al., 2012). However, recent evidence (Yona et al., 2013; Tamoutounour et al., 2013) including this report suggests a contribution by circulating monocytes to the murine tissue macrophage pool. The functional differences between cells derived from these two origins have not been defined. The contemporary view of the mononuclear phagocyte system encompasses several precursor origins including circulating monocytes as precursors of tissue macrophages. Here, we report in humans, two populations of tissue resident cells, CD14$^+$ monocyte-derived macrophages and

Figure 4. Spontaneous Migration of Skin CD14$^+$ Cells Does Not Occur via Lymphatic Vessels

(A) Left panel shows gating strategy used to identify CD14$^+$ cells and CD1c$^+$CD1a$^+$ DCs from live, CD45$^+$, HLA-DR$^+$ cells isolated by digestion (top) and spontaneous migration from skin explants (bottom). Right panel shows relative expression of CCR7 by dermal CD14$^+$ and CD1c$^+$ DC isolated by digestion, migration, and migration in the presence of LPS, TNF-α, and IL-1β. Representative data from at least five donors is shown.

(B) Frequency (as % of HLA-DR$^+$ cells) of dermal CD14$^+$ cells, CD1c$^+$ DCs, CD141$^+$ DCs, and macrophages in skin explant medium after 0–72 hr of culture (n = 6, mean ± SEM). Solid line represents migrated cells, and dotted line represents cells in digested skin remnant.

(C) Pseudocolor immunofluorescence whole-mount microscopy of human skin (T0 = freshly harvested, T48 = 48 hr culture of skin explant ex vivo). Top left panel; T0, depicts distribution of HLA-DR$^+$ (green) and DCSIGN$^+$ (red) cells outside LYVE-1$^+$ lymphatics (blue). Top right panel: DCSIGN$^+$ (red) cells outside LYVE-1$^+$ lymphatics (blue). Lower panel; T48 depicts HLA-DR$^+$ (green) cells mainly located within lymphatic vessels but DCSIGN$^+$ cells are outside the lymphatic vessels. Bottom right; close up three-dimensional reconstruction of boxed area in left panel. Representative image from six donors is shown.

(D) Heat map showing the expression of 96 genes, analyzed by Taqman Low Density Array, by CD14$^+$ cells and CD1c$^+$ DCs isolated by migration and enzymatic skin digestion. Data from five skin donors (pairs indicated by Roman numerals) are shown.

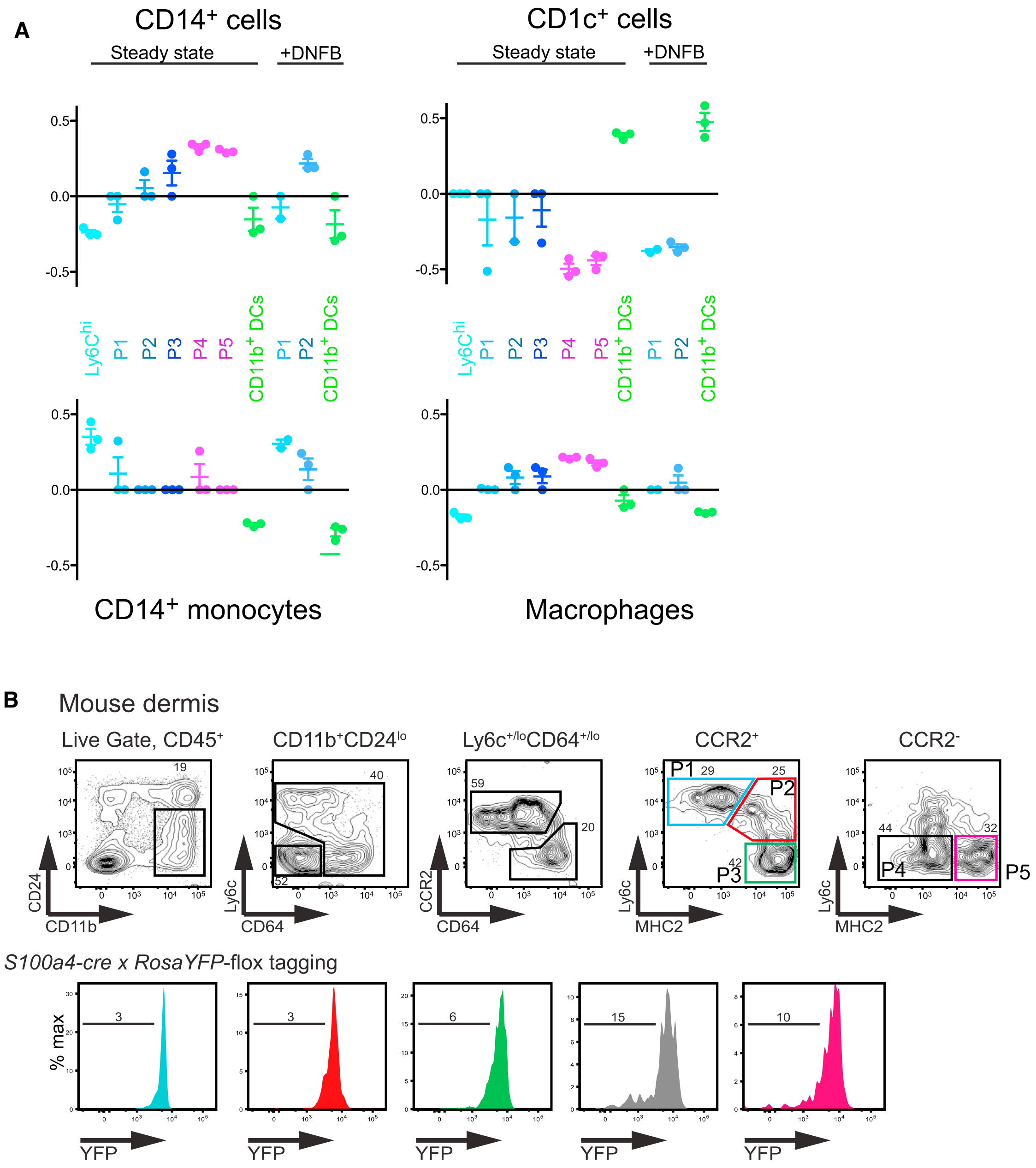

Figure 6. Murine Homolog of Human Dermal CD14$^+$ Cells

(A) CMAP enrichment scores for the signatures of human dermal CD14$^+$ cells, CD1c$^+$ DCs, macrophages, and CD14$^+$ blood monocytes compared with murine CD11b$^+$ dermal cell populations found in steady state and upon contact sensitization with DNFB. P1, tissue monocytes; P2 and P3, dermal monocyte-derived DC-like cells; P4 and P5, dermal macrophages, as described in (Tamoutounour et al., 2013). All enrichment scores were significant at $p < 0.001$.

(B) Flow cytometry analysis of *S100a4xRosaYFP*-flox mouse dermal ear cell suspension. Values in contour plots indicate percentage of cells in the respective gates. %YFP$^-$ cells of the different populations are shown in histogram (lower panel). P1, tissue monocytes; P2 and P3, dermal monocyte-derived DC-like cells; P4 and P5, dermal macrophages; as described in (Tamoutounour et al., 2013). Data shown is representative of six individually analyzed mice from two independent experiments.

fixed macrophages that map to the CD11b$^+$ pool of mouse macrophages. Further work is required to evaluate the long-term contribution of monocytes to the resident macrophage pools.

The functional classification of human tissue CD14$^+$ cells as monocyte-derived macrophages will redirect attention to functional regulation in the skin as opposed to lymph node. A greater understanding of the contribution of monocyte-derived cells in steady state, inflammation, wound healing, and pathology characterized by histiocytosis and granuloma formation will provide further insights into exploiting their origin and functional properties for clinical therapy.

EXPERIMENTAL PROCEDURES

Cell Isolation and Culture

Human samples were obtained in accordance with a favorable ethical opinion from Newcastle, Singapore SingHealth, and National Health Care Group Research Ethics Committees. Normal skin was obtained from mammoplasty and breast reconstruction surgery and digested whole (1 × 1 cm^2) as previously described (Haniffa et al., 2012) to obtain single-cell suspension. Migrating cells were collected from whole skin pieces (1 × 1 cm^2) cultured in RPMI with 10% FCS, and analyzed at serial time points between 0 and 72 hr. Viability was >90% by DAPI exclusion (Sigma). Where stated, 100,000 CFSE-labeled $CD14^+$ blood monocytes were cultured together with 1 × 1cm^2 skin piece during collagenase digestion. Shave biopsies were performed on HSC transplant patients with a DermaBlade® (Shuco). Whole single-cell suspensions were then immunostained and analyzed by flow cytometry. Peripheral blood mononuclear cells were isolated by density centrifugation (Lymphoprep; GE Healthcare). Blood and dermal DC subsets, naive and bulk $CD4^+$ T cells were isolated to >91% purity by fluorescence-activated cell sorting (FACS) using a FACSAriaII and FACSFusion (Becton Dickinson [BD]).

Flow Cytometry

Flow cytometry was performed on a BDLSRII, BDFortessa, and FACSCanto, and data were analyzed with FlowJo (Treestar). Antibodies used are listed in Supplemental Experimental Procedures.

Microscopy

Whole-mount immunofluorescence staining was performed with a previously described protocol (Wang et al., 2014). We fixed a 200 μm skin sheet in PBS containing 2% paraformaldehyde and 30% sucrose overnight at 4°C. Skin was incubated overnight in PBS containing 0.5% BSA and 0.3% Triton X-100 before staining with primary and secondary antibodies at 4°C overnight at each stage. Antibodies used are listed in Supplemental Experimental Procedures. After staining, tissue samples were immersed in VECTASHIELD mounting medium with DAPI (Vector Laboratories). Specimens were viewed using Axio Imager.Z2 fluorescence microscope with Axiovision software v4.8 and Axiocam MR3 camera (Carl Zeiss, Inc.) or Leica Leica TCS SP2 UV confocal microscope and LCS V 2.51 imaging software (Leica). Three-dimensional reconstruction was performed with Imaris7.6.2 soft- ware (www.bitplane.com).

T Cell Alloreaction and Cytokine Production Assays

We cultured 5,000 FACS-sorted dermal DC subsets with 100,000 CFSE-labeled allogeneic naive ($CD4^+CD25^-CCR7^+CD45RO^-$) T cells in U-bottomed 96-well plate. Proliferation was measured by CFSE dilution on day 6. For memory T cell stimulation, autologous blood $CD4^+$ T cells were used to measure recall memory response to *Candida albicans*. We pulsed 5,000 FACS-sorted dermal DC subsets with *Candida albicans* overnight, and cultured them with 100,000 CFSE-labeled (Invitrogen) autologous blood $CD4^+$ T cells on the following day. Media was replenished as necessary throughout the duration of the culture. Cytokine production was measured on day 10, upon stimulation with 20 ng/ml PMA (Sigma-Aldrich; Sigma) and 500 ng/ml Ionomycin (Sigma) for 5 hr in the presence of 10 μg/ml Brefeldin A (Sigma-Aldrich) for the last 2 hr. Cells were fixed and permeabilized (eBioscience Fix/Perm Buffer Set) to allow intracellular cytokine staining. Viaprobe staining (Becton Dickinson; BD) was performed prior to cell fixation to distinguish viable cells.

Blood Monocyte Coculture with HUVECs and Dermal Fibroblasts

HUVECs were cultured in Endothelial Cell Basal Medium 2 (Promo Cell) with the following supplements (FCS, endothelial cell growth supplement, epidermal growth factor, Insulin-like growth factor, vascular endothelial growth factor 165, ascorbic acid, heparin, hydrocortisone [Promo cell]). Dermal fibroblasts were cultured in RPMI with 20% FCS, 1000 u/ml Penicillin and Streptomycin and 2mM L-Glutamine. HUVECs or fibroblasts (30,000/well) were seeded in a 24-well plate and cultured in their respective media (500 μl) for 18 hr at 37°C, 5% CO_2. FACS-purified blood monocyte subsets were added to HUVECs or fibroblast cell cultures (50,000 monocytes/well to a final volume 1 ml) and analyzed on days 1 and 3 after coculture.

Trucount Processing and Analysis

Absolute whole-blood leukocyte analysis was performed as described previously (Jardine et al., 2013). Briefly, 100 μl of whole blood was transferred directly to Trucount tubes (BD). Antibodies were added directly and staining was performed at RT for 20 min, followed by red blood cell lysis by adding 900 μl of BD PharmLyse reagent for 10 min at RT. Samples were then analyzed directly by flow-cytometry. Absolute number of cells per microliters of blood was calculated according to the manufacturer's protocol.

Mouse Dermal Skin Cells Preparation

S100a4-cre mice were purchased from Jackson Laboratory and crossed to R26-stop-EYFP mice in house. Animals positive for the R26-stop-EYFP-flox construct were used for the experiment. Only sex (female) and aged (6–8 weeks) matched mice were used. Mouse skin cells were isolated as described previously (Ginhoux et al., 2007). Briefly, mouse ears were split into dorsal and ventral halves and floated in RPMI-1640 medium (Sigma) containing 1 mg/ml dispase (Invitrogen) for 60 min to allow separation of epidermal and dermal sheets. Dermal sheets were then cut into small pieces and incubated in RPMI containing 10% serum and 0.8 mg/ml collagenase type IV (Worthington-Biochem; 250 U/mg) for 2 hr. Cells were then passed through 19 G syringe and filtered through 100 uM cell strainer (BD Falcon) to obtain a homogenous cell suspension.

Quantitative Real-Time PCR

Total RNA was extracted using the RNeasy Micro Kit (QIAGEN) and treated with DNase I according to manufacturers' instructions (QIAGEN). RNA was used as a template for complementary DNA (cDNA) synthesis using the RevertAid H Minus First Strand cDNA Synthesis Kit with the manufacturers' protocol (Thermo Scientific, Fisher Scientific UK). Real-time PCR was performed with TaqMan Gene-Expression Master Mix and recommended TaqMan Gene-Expression Assays according to manufacturers' instructions (Life Technologies). Reactions were performed using an ABI 7900HT Fast Real-Time PCR System with the instrument's default settings for a standard run. Relative quantification of the messenger RNA (mRNA) amounts was performed using the ΔC_T method and *glyceraldehyde-3-phosphate dehydrogenase* (*GAPDH*) as the reference gene. Taqman assay ID and sequence details are available in Supplemental Experimental Procedures. Details of transcriptomics analysis are available in Supplemental Experimental Procedures.

Statistical Analyses

All statistical analyses were performed using Prism 5.0 (GraphPad Software). All p values are two-tailed using Mann-Whitney U test.

ACCESSION NUMBERS

The microarray data used in these studies are available in the Gene Expression Omnibus (GEO) database (http://www.ncbi.nlm.nih.gov/gds) under the accession numbers GSE35459, GSE60317, GSE49358, and the ImmGen database (www.immgen.org).

SUPPLEMENTAL INFORMATION

Supplemental Information includes two figures, two tables, and Supplemental Experimental Procedures and can be found with this article online at http://dx.doi.org/10.1016/j.immuni.2014.08.006.

AUTHOR CONTRIBUTIONS

M.H., N.M., A. Schlitzer, M.G., M.C., and F.G., conceived the study and designed the experiments. M.H., N.M., M.G., A. Schlitzer, L.J., A. Shin, S.P., E.P., X.-n.W, R.D., K.B., S.C., V.B., and P.M. performed the experiments and analyzed the data. M.H., M.G., N.M., F.G., and M.C. wrote the manuscript. A.L. provided intellectual input. K.D., P.S.W., K.G., and M.P. contributed to the bioinformatics analysis and manuscript preparation. D.M.S commented on the manuscript.

ACKNOWLEDGMENTS

This work was supported by: The Wellcome Trust, UK (WT088555MA; M.H. and N.M.); British Skin Foundation (M.H. and M.G.); Singapore Immunology Network core grant (F.G.); Leukaemia and Lymphoma Research UK (S.P.); Histiocytosis Association and Histiocytosis Research Trust (M.C. and P.M.) and AXA Research Fund (M.C. and M.G.). We thank the following for assistance: D. McDonald and A. Fuller at the Flow Cytometry Core Facility of the Faculty of Medicine Newcastle University; T. Booth and A. Laude of the Bioimaging Unit, Newcastle University; I. Low and N. Binte Shadan of the Flow Cytometry Core Service, Singapore Immunology Network and D. Lilic, and F. Ahmad, Newcastle University for reagents. We thank the Departments of Dermatology, Plastic Surgery and Haematology, Newcastle upon Tyne NHS Trust for clinical material.

Received: May 29, 2014
Accepted: August 14, 2014
Published: September 4, 2014

REFERENCES

Angel, C.E., George, E., Brooks, A.E., Ostrovsky, L.L., Brown, T.L., and Dunbar, P.R. (2006). Cutting edge: CD1a+ antigen-presenting cells in human dermis respond rapidly to CCR7 ligands. J. Immunol. *176*, 5730–5734.

Autissier, P., Soulas, C., Burdo, T.H., and Williams, K.C. (2010). Immunophenotyping of lymphocyte, monocyte and dendritic cell subsets in normal rhesus macaques by 12-color flow cytometry: clarification on DC heterogeneity. J. Immunol. Methods *360*, 119–128.

Bachem, A., Güttler, S., Hartung, E., Ebstein, F., Schaefer, M., Tannert, A., Salama, A., Movassaghi, K., Opitz, C., Mages, H.W., et al. (2010). Superior antigen cross-presentation and XCR1 expression define human CD11c+CD141+ cells as homologues of mouse CD8+ dendritic cells. J. Exp. Med. *207*, 1273–1281.

Bigley, V., Haniffa, M., Doulatov, S., Wang, X.N., Dickinson, R., McGovern, N., Jardine, L., Pagan, S., Dimmick, I., Chua, I., et al. (2011). The human syndrome of dendritic cell, monocyte, B and NK lymphoid deficiency. J. Exp. Med. *208*, 227–234.

Bogunovic, M., Ginhoux, F., Helft, J., Shang, L., Hashimoto, D., Greter, M., Liu, K., Jakubzick, C., Ingersoll, M.A., Leboeuf, M., et al. (2009). Origin of the lamina propria dendritic cell network. Immunity *31*, 513–525.

Caux, C., Vanbervliet, B., Massacrier, C., Dezutter-Dambuyant, C., de Saint-Vis, B., Jacquet, C., Yoneda, K., Imamura, S., Schmitt, D., and Banchereau, J. (1996). CD34+ hematopoietic progenitors from human cord blood differentiate along two independent dendritic cell pathways in response to GM-CSF+TNF alpha. J. Exp. Med. *184*, 695–706.

Chen, C.J., Kono, H., Golenbock, D., Reed, G., Akira, S., and Rock, K.L. (2007). Identification of a key pathway required for the sterile inflammatory response triggered by dying cells. Nat. Med. *13*, 851–856.

Chomarat, P., Banchereau, J., Davoust, J., and Palucka, A.K. (2000). IL-6 switches the differentiation of monocytes from dendritic cells to macrophages. Nat. Immunol. *1*, 510–514.

Chu, C.-C., Ali, N., Karagiannis, P., Di Meglio, P., Skowera, A., Napolitano, L., Barinaga, G., Grys, K., Sharif-Paghaleh, E., Karagiannis, S.N., et al. (2012). Resident CD141 (BDCA3)+ dendritic cells in human skin produce IL-10 and induce regulatory T cells that suppress skin inflammation. J. Exp. Med. *209*, 935–945.

Crozat, K., Guiton, R., Contreras, V., Feuillet, V., Dutertre, C.A., Ventre, E., Vu Manh, T.P., Baranek, T., Storset, A.K., Marvel, J., et al. (2010). The XC chemokine receptor 1 is a conserved selective marker of mammalian cells homologous to mouse CD8alpha+ dendritic cells. J. Exp. Med. *207*, 1283–1292.

Dai, X.M., Ryan, G.R., Hapel, A.J., Dominguez, M.G., Russell, R.G., Kapp, S., Sylvestre, V., and Stanley, E.R. (2002). Targeted disruption of the mouse colony-stimulating factor 1 receptor gene results in osteopetrosis, mononuclear phagocyte deficiency, increased primitive progenitor cell frequencies, and reproductive defects. Blood *99*, 111–120.

de Gruijl, T.D., Sombroek, C.C., Lougheed, S.M., Oosterhoff, D., Buter, J., van den Eertwegh, A.J., Scheper, R.J., and Pinedo, H.M. (2006). A postmigrational switch among skin-derived dendritic cells to a macrophage-like phenotype is predetermined by the intracutaneous cytokine balance. J. Immunol. *176*, 7232–7242.

Gautier, E.L., Shay, T., Miller, J., Greter, M., Jakubzick, C., Ivanov, S., Helft, J., Chow, A., Elpek, K.G., Gordonov, S., et al.; Immunological Genome Consortium (2012). Gene-expression profiles and transcriptional regulatory pathways that underlie the identity and diversity of mouse tissue macrophages. Nat. Immunol. *13*, 1118–1128.

Ginhoux, F., Collin, M.P., Bogunovic, M., Abel, M., Leboeuf, M., Helft, J., Ochando, J., Kissenpfennig, A., Malissen, B., Grisotto, M., et al. (2007). Blood-derived dermal langerin+ dendritic cells survey the skin in the steady state. J. Exp. Med. *204*, 3133–3146.

Hambleton, S., Salem, S., Bustamante, J., Bigley, V., Boisson-Dupuis, S., Azevedo, J., Fortin, A., Haniffa, M., Ceron-Gutierrez, L., Bacon, C.M., et al. (2011). IRF8 mutations and human dendritic-cell immunodeficiency. N. Engl. J. Med. *365*, 127–138.

Han, B., Luo, G., Shi, Z.Z., Barrios, R., Atwood, D., Liu, W., Habib, G.M., Sifers, R.N., Corry, D.B., and Lieberman, M.W. (2002). Gamma-glutamyl leukotrienase, a novel endothelial membrane protein, is specifically responsible for leukotriene D(4) formation in vivo. Am. J. Pathol. *161*, 481–490.

Haniffa, M., Ginhoux, F., Wang, X.N., Bigley, V., Abel, M., Dimmick, I., Bullock, S., Grisotto, M., Booth, T., Taub, P., et al. (2009). Differential rates of replacement of human dermal dendritic cells and macrophages during hematopoietic stem cell transplantation. J. Exp. Med. *206*, 371–385.

Haniffa, M., Shin, A., Bigley, V., McGovern, N., Teo, P., See, P., Wasan, P.S., Wang, X.-N., Malinarich, F., Malleret, B., et al. (2012). Human tissues contain CD141hi cross-presenting dendritic cells with functional homology to mouse CD103+ nonlymphoid dendritic cells. Immunity *37*, 60–73.

Haniffa, M., Collin, M., and Ginhoux, F. (2013). Ontogeny and functional specialization of dendritic cells in human and mouse. Adv. Immunol. *120*, 1–49.

Hashimoto, D., Chow, A., Noizat, C., Teo, P., Beasley, M.B., Leboeuf, M., Becker, C.D., See, P., Price, J., Lucas, D., et al. (2013). Tissue-resident macrophages self-maintain locally throughout adult life with minimal contribution from circulating monocytes. Immunity *38*, 792–804.

Hoeffel, G., Wang, Y., Greter, M., See, P., Teo, P., Malleret, B., Leboeuf, M., Low, D., Oller, G., Almeida, F., et al. (2012). Adult Langerhans cells derive predominantly from embryonic fetal liver monocytes with a minor contribution of yolk sac-derived macrophages. J. Exp. Med. *209*, 1167–1181.

Jakubzick, C., Gautier, E.L., Gibbings, S.L., Sojka, D.K., Schlitzer, A., Johnson, T.E., Ivanov, S., Duan, Q., Bala, S., Condon, T., et al. (2013). Minimal differentiation of classical monocytes as they survey steady-state tissues and transport antigen to lymph nodes. Immunity *39*, 599–610.

Jardine, L., Barge, D., Ames-Draycott, A., Pagan, S., Cookson, S., Spickett, G., Haniffa, M., Collin, M., and Bigley, V. (2013). Rapid detection of dendritic cell and monocyte disorders using CD4 as a lineage marker of the human peripheral blood antigen-presenting cell compartment. Front Immunol *4*, 495.

Jongbloed, S.L., Kassianos, A.J., McDonald, K.J., Clark, G.J., Ju, X., Angel, C.E., Chen, C.J., Dunbar, P.R., Wadley, R.B., Jeet, V., et al. (2010). Human CD141+ (BDCA-3)+ dendritic cells (DCs) represent a unique myeloid DC subset that cross-presents necrotic cell antigens. J. Exp. Med. *207*, 1247–1260.

Klechevsky, E., Morita, R., Liu, M., Cao, Y., Coquery, S., Thompson-Snipes, L., Briere, F., Chaussabel, D., Zurawski, G., Palucka, A.K., et al. (2008). Functional specializations of human epidermal Langerhans cells and CD14+ dermal dendritic cells. Immunity *29*, 497–510.

Langlet, C., Tamoutounour, S., Henri, S., Luche, H., Ardouin, L., Grégoire, C., Malissen, B., and Guilliams, M. (2012). CD64 expression distinguishes monocyte-derived and conventional dendritic cells and reveals their distinct role during intramuscular immunization. J. Immunol. *188*, 1751–1760.

Lenz, A., Heine, M., Schuler, G., and Romani, N. (1993). Human and murine dermis contain dendritic cells. Isolation by means of a novel method and phenotypical and functional characterization. J. Clin. Invest. *92*, 2587–2596.

Matthews, K., Chung, N.P., Klasse, P.J., Moore, J.P., and Sanders, R.W. (2012). Potent induction of antibody-secreting B cells by human dermal-derived CD14+ dendritic cells triggered by dual TLR ligation. J. Immunol. *189*, 5729–5744.

McKenna, H.J., Stocking, K.L., Miller, R.E., Brasel, K., De Smedt, T., Maraskovsky, E., Maliszewski, C.R., Lynch, D.H., Smith, J., Pulendran, B., et al. (2000). Mice lacking flt3 ligand have deficient hematopoiesis affecting hematopoietic progenitor cells, dendritic cells, and natural killer cells. Blood *95*, 3489–3497.

Miller, J.C., Brown, B.D., Shay, T., Gautier, E.L., Jojic, V., Cohain, A., Pandey, G., Leboeuf, M., Elpek, K.G., Helft, J., et al.; Immunological Genome Consortium (2012). Deciphering the transcriptional network of the dendritic cell lineage. Nat. Immunol. *13*, 888–899.

Morelli, A.E., Rubin, J.P., Erdos, G., Tkacheva, O.A., Mathers, A.R., Zahorchak, A.F., Thomson, A.W., Falo, L.D.J., Jr., and Larregina, A.T. (2005). CD4+ T cell responses elicited by different subsets of human skin migratory dendritic cells. J. Immunol. *175*, 7905–7915.

Nestle, F.O., Zheng, X.G., Thompson, C.B., Turka, L.A., and Nickoloff, B.J. (1993). Characterization of dermal dendritic cells obtained from normal human skin reveals phenotypic and functionally distinctive subsets. J. Immunol. *151*, 6535–6545.

Ohl, L., Mohaupt, M., Czeloth, N., Hintzen, G., Kiafard, Z., Zwirner, J., Blankenstein, T., Henning, G., and Förster, R. (2004). CCR7 governs skin dendritic cell migration under inflammatory and steady-state conditions. Immunity *21*, 279–288.

Penel-Sotirakis, K., Simonazzi, E., Péguet-Navarro, J., and Rozières, A. (2012). Differential capacity of human skin dendritic cells to polarize CD4+ T cells into IL-17, IL-21 and IL-22 producing cells. PLoS ONE *7*, e45680.

Plantinga, M., Guilliams, M., Vanheerswynghels, M., Deswarte, K., Branco-Madeira, F., Toussaint, W., Vanhoutte, L., Neyt, K., Killeen, N., Malissen, B., et al. (2013). Conventional and monocyte-derived CD11b(+) dendritic cells initiate and maintain T helper 2 cell-mediated immunity to house dust mite allergen. Immunity *38*, 322–335.

Poulin, L.F., Salio, M., Griessinger, E., Anjos-Afonso, F., Craciun, L., Chen, J.L., Keller, A.M., Joffre, O., Zelenay, S., Nye, E., et al. (2010). Characterization of human DNGR-1+ BDCA3+ leukocytes as putative equivalents of mouse CD8alpha+ dendritic cells. J. Exp. Med. *207*, 1261–1271.

Randolph, G.J., Beaulieu, S., Lebecque, S., Steinman, R.M., and Muller, W.A. (1998). Differentiation of monocytes into dendritic cells in a model of transendothelial trafficking. Science *282*, 480–483.

Robbins, S.H., Walzer, T., Dembélé, D., Thibault, C., Defays, A., Bessou, G., Xu, H., Vivier, E., Sellars, M., Pierre, P., et al. (2008). Novel insights into the relationships between dendritic cell subsets in human and mouse revealed by genome-wide expression profiling. Genome Biol. *9*, R17.

Schlitzer, A., McGovern, N., Teo, P., Zelante, T., Atarashi, K., Low, D., Ho, A.W., See, P., Shin, A., Wasan, P.S., et al. (2013). IRF4 transcription factor-dependent CD11b+ dendritic cells in human and mouse control mucosal IL-17 cytokine responses. Immunity *38*, 970–983.

Schulz, C., Gomez Perdiguero, E., Chorro, L., Szabo-Rogers, H., Cagnard, N., Kierdorf, K., Prinz, M., Wu, B., Jacobsen, S.E., Pollard, J.W., et al. (2012). A lineage of myeloid cells independent of Myb and hematopoietic stem cells. Science *336*, 86–90.

Segura, E., Touzot, M., Bohineust, A., Cappuccio, A., Chiocchia, G., Hosmalin, A., Dalod, M., Soumelis, V., and Amigorena, S. (2013). Human inflammatory dendritic cells induce Th17 cell differentiation. Immunity *38*, 336–348.

Steinman, R.M. (2007). Lasker Basic Medical Research Award. Dendritic cells: versatile controllers of the immune system. Nat. Med. *13*, 1155–1159.

Stoitzner, P., Zanella, M., Ortner, U., Lukas, M., Tagwerker, A., Janke, K., Lutz, M.B., Schuler, G., Echtenacher, B., Ryffel, B., et al. (1999). Migration of langerhans cells and dermal dendritic cells in skin organ cultures: augmentation by TNF-alpha and IL-1beta. J. Leukoc. Biol. *66*, 462–470.

Tamoutounour, S., Henri, S., Lelouard, H., de Bovis, B., de Haar, C., van der Woude, C.J., Woltman, A.M., Reyal, Y., Bonnet, D., Sichien, D., et al. (2012). CD64 distinguishes macrophages from dendritic cells in the gut and reveals the Th1-inducing role of mesenteric lymph node macrophages during colitis. Eur. J. Immunol. *42*, 3150–3166.

Tamoutounour, S., Guilliams, M., Montanana Sanchis, F., Liu, H., Terhorst, D., Malosse, C., Pollet, E., Ardouin, L., Luche, H., Sanchez, C., et al. (2013). Origins and functional specialization of macrophages and of conventional and monocyte-derived dendritic cells in mouse skin. Immunity *39*, 925–938.

van Furth, R., and Cohn, Z.A. (1968). The origin and kinetics of mononuclear phagocytes. J. Exp. Med. *128*, 415–435.

Varol, C., Vallon-Eberhard, A., Elinav, E., Aychek, T., Shapira, Y., Luche, H., Fehling, H.J., Hardt, W.D., Shakhar, G., and Jung, S. (2009). Intestinal lamina propria dendritic cell subsets have different origin and functions. Immunity *31*, 502–512.

Wang, X.N., McGovern, N., Gunawan, M., Richardson, C., Windebank, M., Siah, T.W., Lim, H.Y., Fink, K., Li, J.L., Ng, L.G., et al. (2014). A three-dimensional atlas of human dermal leukocytes, lymphatics, and blood vessels. J. Invest. Dermatol. *134*, 965–974.

Watchmaker, P.B., Lahl, K., Lee, M., Baumjohann, D., Morton, J., Kim, S.J., Zeng, R., Dent, A., Ansel, K.M., Diamond, B., et al. (2014). Comparative transcriptional and functional profiling defines conserved programs of intestinal DC differentiation in humans and mice. Nat. Immunol. *15*, 98–108.

Yona, S., Kim, K.W., Wolf, Y., Mildner, A., Varol, D., Breker, M., Strauss-Ayali, D., Viukov, S., Guilliams, M., Misharin, A., et al. (2013). Fate mapping reveals origins and dynamics of monocytes and tissue macrophages under homeostasis. Immunity *38*, 79–91.

Yoshida, H., Hayashi, S., Kunisada, T., Ogawa, M., Nishikawa, S., Okamura, H., Sudo, T., Shultz, L.D., and Nishikawa, S. (1990). The murine mutation osteopetrosis is in the coding region of the macrophage colony stimulating factor gene. Nature *345*, 442–444.

Zaba, L.C., Fuentes-Duculan, J., Steinman, R.M., Krueger, J.G., and Lowes, M.A. (2007). Normal human dermis contains distinct populations of CD11c+BDCA-1+ dendritic cells and CD163+FXIIIA+ macrophages. J. Clin. Invest. *117*, 2517–2525.

Zigmond, E., Varol, C., Farache, J., Elmaliah, E., Satpathy, A.T., Friedlander, G., Mack, M., Shpigel, N., Boneca, I.G., Murphy, K.M., et al. (2012). Ly6C hi monocytes in the inflamed colon give rise to proinflammatory effector cells and migratory antigen-presenting cells. Immunity *37*, 1076–1090.

Immunity, Volume *41*

Supplemental Information

Human Dermal CD14$^+$ Cells Are a Transient Population of Monocyte-Derived Macrophages

Naomi McGovern, Andreas Schlitzer, Merry Gunawan, Laura Jardine, Amanda Shin, Elizabeth Poyner, Kile Green, Rachel Dickinson, Xiao-nong Wang, Donovan Low, Katie Best, Samuel Covins, Paul Milne, Sarah Pagan, Khadija Aljefri, Martin Windebank, Diego Miranda Saavedra, Pavandip Singh Wasan, Duan Kaibo, Michael Poidinger, Venetia Bigley, Florent Ginhoux, Matthew Collin, and Muzlifah Haniffa

Supplemental Experimental Procedures

Microarray analysis

Microarray data from purified dermal macrophages, epidermal LCs and $CD14^+$ cells hybridized on Illumina Human WG-6 BeadChip were integrated with GSE35457 Illumina HumanHT-12 V4.0 microarray data of human blood and skin monocyte and DC subsets. The integrated data were quantile normalized without background correction. Tissue specific probes were determined with samples from GSE35457 dataset using a t-test step similar to that previously described (Haniffa et al., 2012) but without using the Tissue-specific Gene Expression and Regulation (TiGER) database (Liu et al., 2008). Tissue specific probes were removed from the integrated data. Finally, ComBat (Johnson et al., 2007) was used to correct the batch effect observed in the integrated data. Signature genes for each cell subtype were identified by comparing one subtype versus other subtypes pooled using t-test and selected with Benjamini-Hochberg (BH) multiple testing (Benjamini and Hochberg, 1995) corrected p-value of < 0.05 and fold-change of > 1.5.

To identify the mouse homolog of dermal $CD14^+$ cells, human APC subsets were compared with mouse APC subsets in mouse microarray data GSE49358 (Affymetrix Mouse Gene 1.0 ST array). Mouse array data was processed using Bioconductor *oligo* pacakge (http://www.bioconductor.org) with quantile normalization and gene level expression value summarization using only the core probes. For genes with multiple transcript clusters mapping, the average expression values of the transcript clusters were used. For comparison of human APC subsets to mouse APC subsets, signatures of human APC subsets were first converted to mouse signatures by using HomoloGene table build 67 (http://www.ncbi.nlm.nih.gov/homologene/) for ortholog gene mapping. Enrichment scores were then computed on the mouse APC subsets with the converted mouse signatures using connectivity map (CMAP) analysis as previously described (Haniffa et al., 2012).

For comparative analysis of genes defining monocyte-macrophages in contrast to DCs between human and mouse, the up-regulated and down-regulated genes comparing monocyte-mocrophages to myeloid DC subsets were identified in human array data and mouse array data separately. For human array data, the monocyte, macrophage, and $CD14^+$ DC samples were pooled and compared to other DC subsets. For mouse data, monocyte, macrophage and DC subsets from the ImmGen data GSE15907 ($DC.8^+$ Sp, $DC4^+$ Sp, $DC.8^+$ SLN, $DC.4^+$ SLN,

DC.IIhilang^{+}103^{+}11blo.SLN, DC.IIhilang^{-}103^{-}11b^{+}.SLN, DC.103^{+}11b^{-}.Lu, DC.103^{-}11b^{+}24^{+}.Lu, Mo.6C^{+}II^{-}.Bl, Mo.6C^{+}II^{+}.Bl, Mo.6C^{-}II^{-}.Bl, Mo.6C^{-}II^{+}.Bl, Mo.6C^{-}IIint.Bl, MF.103^{-}11b^{+}24^{-}, MF.Lu and MF.II^{-}480hi.PC, hybridised using Affymetrix Mouse Gene 1.0 ST array) and GSE49358 (P4 and P5 dermal macrophage subsets) were extracted and together processed similarly as processing GSE49358 in the comparative analysis of APC subsets between human and mouse described earlier. Monocyte and macrophage subsets as one group were compared with DC subsets as another group. For both human data and mouse data, the differentially expressed genes (DEGs) were identified using t-test with BH multiple testing corrected p-value of <0.05 and fold-change of >1.5. The NCBI HomoloGene table (build 67) was used to map the DEGs between human and mouse. Only DEGs with one-to-one ortholog mapping between the two species were kept. Pearson correlation analysis was done to compare the log fold-change values of the conserved human and mouse DEGs for monocytes and macrophages compared to DCs (*r=0.58* and *p-value = 2.2E-16)*.

All the data processing and analyses were carried out using the R programming language version 2.12.2 (http://www.r-project.org) and Pipeline Pilot (http://accelrys.com/products/pipeline-pilot/)

Antibodies used for flow cytometry and microscopy

The following antibodies were used for flow cytometry: CD45 v500 (H130, BD Horizon), HLA-DR percpcy5.5 (LN3, Ebioscience), HLA-DR PE (L243, ebioscience), CD14 APCCy7 (61D3, Ebioscience), CD1a A700 (HI149, Biolegend), CD1c Pecy7 (L161, Biolegend). CD11c v450 (B-ly6, BD Pharmingen), CD141 APC (AD5-14H12, Miltenyi), DC-SIGN PE (DCS-8CI, Biolegend), CD163 PE (215927, R&D), BTLA PE (MIH26, Biolegend), CD11b PE (ICRF44, Biolegend), SIRP1a PE (SESA5, Biolegend), CX3CR1 PE (2A9-1, Biolegend), CD26 PE (BA5b, Biolegend), CD64 PE (10.1, Biolegend), IRF4 PE (M-17, Sant Cruz), FLT3 PE (4G8, BD Pharmingen), MCSFR PE (12-3A3-1B10, Ebioscience). The following antibodies were used for microscopy: CD11c (B-ly6, BD Biosciences), CD11c FITC (BU15, AbD seotec) HLA-DR FITC (L243, BD Biosciences), LYVE1 (Polyclonal, R&D), FXIIIa (polyclonal, Enzyme Research Lab), DCSIGN (5D7, Abcam); donkey anti-rabbit and donkey anti-mouse Dy488, Dy549 or Dy649 (Jackson ImmunoResearch) and donkey anti-goat and donkey anti-sheep AlexaFluor647 or AlexaFluor488 (Invitrogen). Antibodies used for mouse analysis were purchased from BD, eBioscience and R&D: CD14

(Sa14-2), CD11b (M1/70), CD24 (M1/69), CD45 (30-F11), CD64 (X54-5/7.1), MHC2 (M5/114.15.2), CCR2 (475301) and Ly6C (HK1.4).

RQ-PCR primer details

Human Gene	Assay ID	RefSeq	Exon Boundary	Assay Location	Amplicon Length
GAPDH	4352934E	NM_002046.3	3-4	Not Given	122
LYVE1	Hs00272659_m1	NM_006691.3	1-2	373	68
CD209	Hs01588349_m1	NM_021155.3	6-7	1078	131
ZBTB46	Hs01008168_m1	NM_025224.3	4-5	1595	59
F13A1	Hs00173388_m1	NM_000129.3	2-3	254	80
GGT5	Hs00269779_m1	NM_001099781.1	11-12	2036	63
IL1A	Hs00174092_m1	NM_000575.3	6-7	1578	69

Figure S1, related to Figure 3

A and B. Ingenuity™ Pathway Analysis (IPA) of the differentially expressed genes (DEGs) comparing monocytes, macrophages and $CD14^+$ cells (monocyte-macrophage group) to $CD141^+$ DCs and $CD1c^+$ DCs (DC group). DEGs were identified using t-test with BH multiple testing corrected for p-value <0.05 and fold change >1.5 between the two groups. Using these criteria, 301 up-regulated and 278 down-regulated genes (monocyte-macrophage group vs DC group) were identified. The graphs show enriched pathways for the up and down regulated genes. Blue bars indicate the p values (-log10) for pathway enrichment. The yellow squares indicate the ratio of the number of up or down-regulated genes mapped to the enriched pathway to the total number of molecules on that pathway represented by the dashed yellow line. The horizontal solid yellow line corresponds to the p-value = 0.05 threshold.

Figure S1

A Upregulated pathways in human monocytes and macrophages compared to DCs

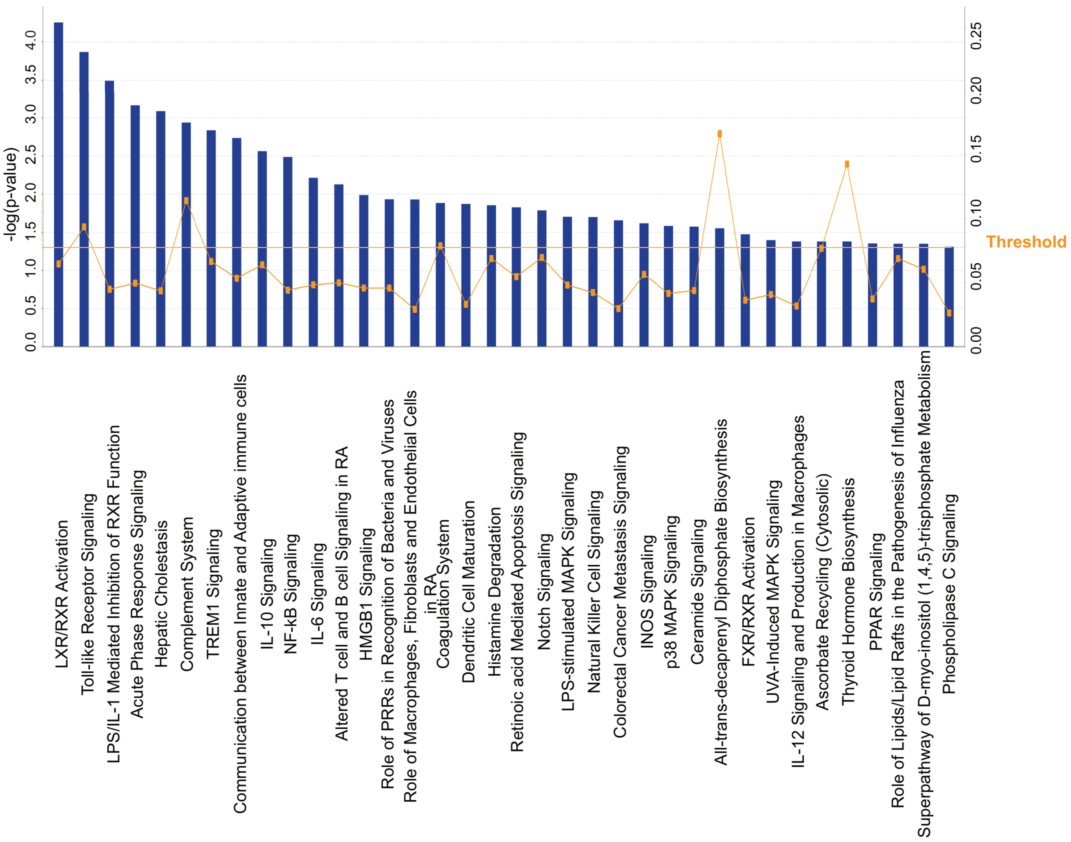

B Downregulated pathways in human monocytes and macrophages compared to DCs

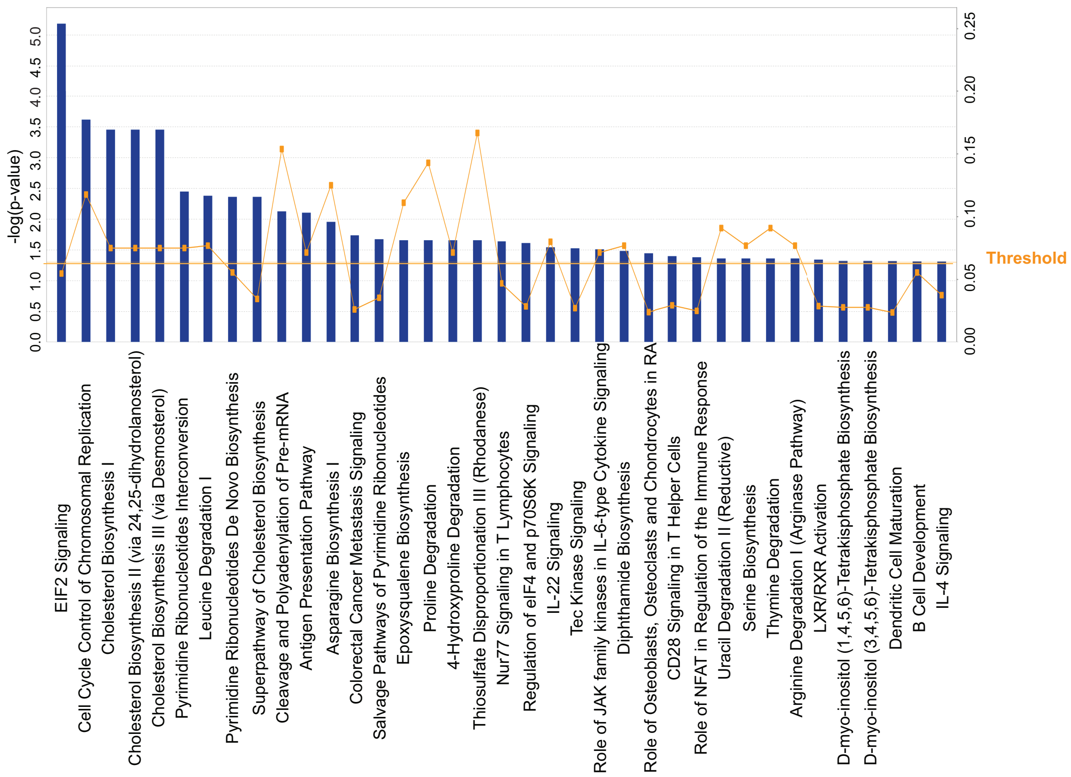

Figure S2, related to Figure 4

A. Overlay histogram showing DCSIGN expression analysed by flow cytometry by $CD14^+$ cells (blue) and CD1c+ DCs (green) spontaneously migrated from skin at 24 hr and 48 hr of skin explant culture. Isotype control shown in grey, representative data from n=4 independent donors shown.

B. DCSIGN expression evaluated by fluorescence microscopy analysis of cytospin prepared migrated cells from skin explant cultured for 60 hr. Representative image from n= 3 donors shown.

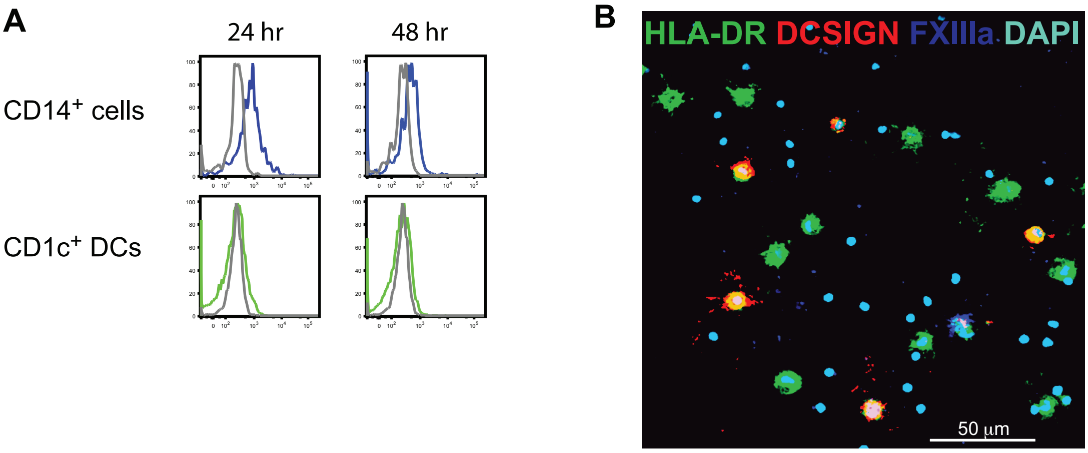

Table S1, related to Figure 2. Clinical data of HSCT patients

Patient	Diagnosis	Donor	Conditioning Regime	Alemtuzumab Dose
1	AML	MUD	FLAMSA	30
2	lymphoma	Sib	Flu Mel	30
3	AML	MUD	FLAMSA	30
4	AML	Sib	FLAMSA	30
5	MDS	MUD	FLAMSA	30
6	AML	MUD	Flu Bu	30
7	lymphoma	Sib	Flu Mel	30
8	lymphoma	MUD	Flu Mel	60
9	ALL	MUD	Flu Mel	60
10	AML	Sib	Flu Bu	30
11	MDS	MUD	Flu Bu	60
12	MDS	MUD	Flu Bu	60
13	lymphoma	MUD	Flu Mel	60
14	AML	MUD	Flu Bu	30
15	MDS	MUD	FLAMSA	30
16	lymphoma	MUD	Flu Mel	60
17	AML	MUD	Flu Bu	60

AML = acute myeloid leukaemia
MDS = myelodysplastic syndrome
MUD = matched unrelated donor
Sib = matched sibling donor
FLAMSA = fludarabine, amsacarine, ara-C
Flu Bu = fludarabine busulphan
Flu Mel = fludarabine melphalan
